# Endothelial cell cycle state determines propensity for arterial-venous fate

**DOI:** 10.1038/s41467-022-33324-7

**Published:** 2022-10-06

**Authors:** Nicholas W. Chavkin, Gael Genet, Mathilde Poulet, Erin D. Jeffery, Corina Marziano, Nafiisha Genet, Hema Vasavada, Elizabeth A. Nelson, Bipul R. Acharya, Anupreet Kour, Jordon Aragon, Stephanie P. McDonnell, Mahalia Huba, Gloria M. Sheynkman, Kenneth Walsh, Karen K. Hirschi

**Affiliations:** 1 Department of Cell Biology, University of Virginia School of Medicine, Charlottesville, VA 22908 USA; 2 Robert M. Berne Cardiovascular Research Center, University of Virginia School of Medicine, Charlottesville, VA 22908 USA; 3 Department of Medicine, Yale Cardiovascular Research Center Yale University School of Medicine, New Haven, CT 06520 USA; 4 Department of Molecular Physiology and Biological Physics, University of Virginia School of Medicine, Charlottesville, VA 22908 USA; 5 Department of Biochemistry and Molecular Genetics, University of Virginia School of Medicine, Charlottesville, VA 22908 USA; 6 Center for Public Health Genomics, University of Virginia School of Medicine, Charlottesville, VA 22908 USA; 7 UVA Comprehensive Cancer Center, University of Virginia, Charlottesville, VA 22908 USA; 8 Hematovascular Biology Center, University of Virginia School of Medicine, Charlottesville, VA 22908 USA

**Keywords:** Angiogenesis, Morphogenesis, Cell division, Angiogenesis, Stem-cell differentiation

## Abstract

During blood vessel development, endothelial cells become specified toward arterial or venous fates to generate a circulatory network that provides nutrients and oxygen to, and removes metabolic waste from, all tissues. Arterial-venous specification occurs in conjunction with suppression of endothelial cell cycle progression; however, the mechanistic role of cell cycle state is unknown. Herein, using Cdh5-CreER^T2^;R26FUCCI2aR reporter mice, we find that venous endothelial cells are enriched for the FUCCI-Negative state (early G1) and BMP signaling, while arterial endothelial cells are enriched for the FUCCI-Red state (late G1) and TGF-β signaling. Furthermore, early G1 state is essential for BMP4-induced venous gene expression, whereas late G1 state is essential for TGF-β1-induced arterial gene expression. Pharmacologically induced cell cycle arrest prevents arterial-venous specification defects in mice with endothelial hyperproliferation. Collectively, our results show that distinct endothelial cell cycle states provide distinct windows of opportunity for the molecular induction of arterial vs. venous fate.

## Abstract (web summary)

During blood vessel development, endothelial cells become specified toward arterial or venous fates. Chavkin et al find that distinct endothelial cell cycle states provide windows of opportunity for the molecular induction of arterial or venous fate.

## Introduction

Healthy tissue development and maintenance require a functional blood circulatory network comprised of arterial, capillary, and venous blood vessels lined with specialized endothelial cells. Acquisition of specialized arterial and venous endothelial cell phenotypes generally occurs in conjunction with suppression of endothelial cell cycle progression^1–4^. However, we lack understanding of mechanisms that coordinately regulate endothelial cell growth suppression and phenotypic specialization during vascular remodeling, which creates significant roadblocks for clinical therapies, tissue engineering and regenerative medicine.

Our previous work showed that a Notch-Cx37-p27 signaling axis promotes endothelial cell cycle arrest that enables the upregulation of arterial genes, which can be activated through shear stress magnitudes typical of arteries and arterioles^2^. However, it is not clear whether a specific state of the cell cycle plays a role in venous endothelial cell specification, or whether distinct cell cycle states control the differential specification of arterial and venous endothelial cells. In this regard, multiple signaling pathways have been implicated in the regulation of arterial–venous network formation, including TGF-β and BMP^5–9^, but how these signaling pathways function in coordination with cell cycle state to induce specific endothelial cell phenotypes is also not known.

To fill these knowledge gaps, we combined the previously generated transgenic alleles of the Cre-responsive Fluorescent Ubiquitination Cell Cycle Indicator (FUCCI) reporter mice labeled R26FUCCI2aR^10^ and endothelial-specific tamoxifen-inducible Cdh5-CreERT2^11^ mice to generate mice that allow visualization of cell cycle state specifically in endothelial cells. The FUCCI reporter system takes advantage of differential ubiquitination and degradation of cell cycle-related proteins Cdt1 and Geminin to identify cell cycle states by expressing fusion proteins mCherry-hCdt1(30/120) and mVenus-hGem(1/110)^10^. In S, G2, and M states, only Geminin is expressed, resulting in mVenus-hGem(1/110) expression and green fluorescence labeling in S/G2/M states; Geminin is then degraded following mitosis. Cdt1 is expressed and mCherry-hCdt1(30/120) accumulates as cells progress through G1 state, and it is then degraded when cells transition into S phase. Of note, in the FUCCI2a mice that we employ in our studies, the reporters do not distinguish between cells in G1 and G0; both express Cdt1 and the red reporter, and the FUCCI-Negative cells are thought to be in an earlier stage of G1^10,12^.

In these studies, using these endothelial-specific FUCCI reporter mice, we demonstrate that endothelial cells in veins/venules vs. arteries/arterioles have a propensity to be in different cell cycle states during vascular development and in adulthood; FUCCI-Negative vs. FUCCI-Red G1, respectively. Interestingly, in embryonic stem cells, these states are molecularly distinct and represent distinct windows of opportunity for the induction of mesoderm/endoderm vs. ectoderm lineages^13,14^. Thus, to gain a broader understanding of endothelial cell cycle state and identity, we performed single-cell RNA sequencing of developing retinal endothelial cells, in combination with bulk RNA sequencing of retinal endothelial cells expressing different FUCCI reporters. Collectively, these data analyses reveal that specification towards venous or arterial phenotypes associates with enrichment of FUCCI-Negative vs. FUCCI-Red G1 states, respectively, at a single cell level. In addition, BMP signaling is enriched in venous and TGF-β signaling enriched in arterial endothelial cells. We then used human umbilical vein endothelial cells transduced with a lentivirus expressing the Fast-FUCCI reporter^15^ (HUVEC-FUCCI) to demonstrate that BMP signaling induces venous gene expression only in early G1; whereas, TGF-β induces arterial gene expression only in late G1. Finally, using Cx37-deficient mice that exhibit endothelial cell hyperproliferation and impaired arterial–venous network development, we show that pharmacological induction of endothelial cell cycle arrest prevents these vascular defects and enables normal arterial–venous network formation. These studies reveal a critical and previously unknown molecular connection between endothelial cell cycle state and fate; specifically, endothelial cell cycle state determines the propensity for arterial vs. venous fate specification.

## Results

### Arterial–venous endothelial cell cycle state

**Fig. 1:**
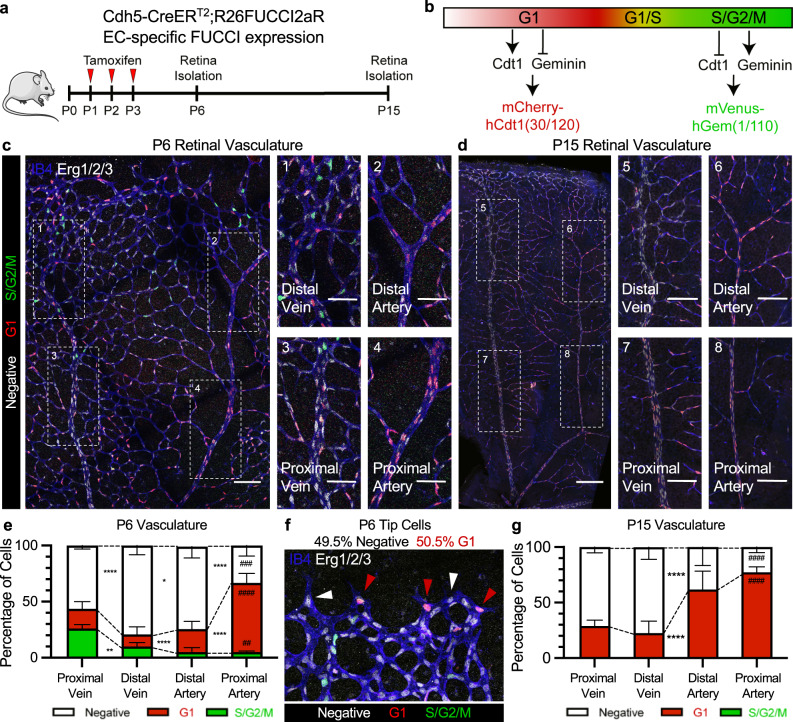
Endothelial cell cycle state during retinal vascular development. **a**, **b** Cdh5-CreER^T2^;R26FUCCI2aR transgenic mouse allows for tamoxifen-induced endothelial cell-specific expression of the FUCCI reporter, which uses dynamic degradation of mVenus-hGem(1/110) and mCherry-hCdt1(30/120) in different cell cycle states to distinguish FUCCI-Negative, FUCCI-Red G1, and FUCCI-Green S/G2/M cell cycle states. **c** Representative confocal image of P6 retinas from Cdh5-CreER^T2^;R26FUCCI2aR mice immunostained with IB4 and anti-Erg1/2/3 (scale bar = 150 μm), 1–4) magnified images of vein and artery branch sections (scale bar = 50 μm). **d** Representative confocal image of P15 retinas from Cdh5-Cre ER^T2^;R26FUCCI2aR mice immunostained with IB4 and anti-Erg1/2/3 (scale bar = 200 μm), 5–8) magnified images of vein and artery branch sections (scale bar = 100 μm). **e** Quantification of cell cycle states in vascular regions of P6 retinal vasculature (mean +/− SD, # shows statistical significance between vein and artery, *n* = 4 retinas, 5 to 10 images per retina). **f** Representative confocal image of tip cells from Cdh5-Cre ER^T2^;R26FUCCI2aR mice immunostained with IB4 and anti-Erk1/2/3 (scale bar = 25 μm). White arrows indicate FUCCI-Negative state and red arrows indicate FUCCI-Red G1 state. Quantification of cell cycle states in endothelial tip cells (*n* = 4 retinas, 20–25 tip cells per retina). **g** Cell cycle states quantified in veins and arteries of P15 retinal vasculature (mean +/− SD, # shows statistical significance between vein and artery, *n* = 4 retinas, 5 to 10 images per retina). Source data are provided as a Source data file. Statistical comparison of means by two-way ANOVA post hoc Tukey, *p*-values represented as */# < 0.05, **/## < 0.01, ***/### < 0.001, ****/#### < 0.0001.

To determine the cell cycle state of endothelial cells during arterial–venous specification, we crossed mice expressing a FUCCI reporter with a flox-stop-flox cassette (R26FUCCI2aR)^10^ with mice expressing the endothelial-specific Cdh5-CreERT2^11^ (Fig. 1a, b). In these tamoxifen-treated mice, at postnatal day (P)6, we examined retinal endothelial cells in the developing arterial–venous network (Fig. 1c). We found that endothelial cells in the remodeling areas closer to venous vessels are enriched for the FUCCI-Green reporter (S/G2/M). Endothelial cells in and near the arterial branches are enriched for the FUCCI-Red reporter (G1); whereas, endothelial cells in and near the venous branches are predominantly FUCCI-Negative. These patterns persisted in P15 retinal vasculature, in which arterial and venous branches have matured, although no endothelial cells in S/G2/M states were found (Fig. 1d).

Quantification of endothelial cell cycle states in the P6 retinal vasculature revealed a transition in endothelial cell cycles states from S/G2/M to G1 in contiguous regions of the vascular plexi (proximal vein, distal vein, distal artery, proximal artery) (Fig. 1e). As endothelial Tip cells have been shown to acquire an arterial phenotype^16,17^, we quantified the percentage of Tip cells in distinct cell cycle states by quantifying cell cycle state in angiogenic leading endothelial cells with notable filopodia extensions. We found 49.5% to be FUCCI-Negative, 50.5% FUCCI-Red G1, and none in S/G2/M (Fig. 1f). In addition, we found that P15 retinal endothelial cells followed the same pattern where a greater proportion of venous endothelial cells were FUCCI-Negative and arterial endothelial cells were enriched for FUCCI-Red G1, with no cells in S/G2/M identified at P15 (Fig. 1g). Over the time-course of retinal vascular development (P0-P18), the percentage of endothelial cells in S/G2/M decreased to undetectable levels by P15, FUCCI-Negative endothelial cells gradually decreased to ~33%, and FUCCI-Red G1 endothelial cells gradually increased to ~66% (Supplementary Fig. 1a). In addition, among capillary endothelial cells, those closer to veins exhibited a higher proportion of FUCCI-Negative cells, whereas those closer to arteries exhibited a higher proportion in FUCCI-Red G1 (Supplementary Fig. 1b, c). A propensity for distinct cell cycle states was also evident in arterial and venous endothelial cells in other tissues of P6 neonates, such as the heart (Supplementary Fig. 1d**)**. In addition, during early development, from embryonic days (E)9.5-E11.5, endothelial cells in the cardinal vein were enriched for FUCCI-Negative; in contrast, endothelial cells in the dorsal aorta were highly enriched for FUCCI-Red G1 (Supplementary Fig. 1e, f). Interestingly, we found that this difference in cell cycle states among venous and arterial endothelial cells, respectively, persisted in adult blood vessels (Supplementary Fig. 1g, h).

### Endothelial cell cycle-dependent gene expression

**Fig. 2:**
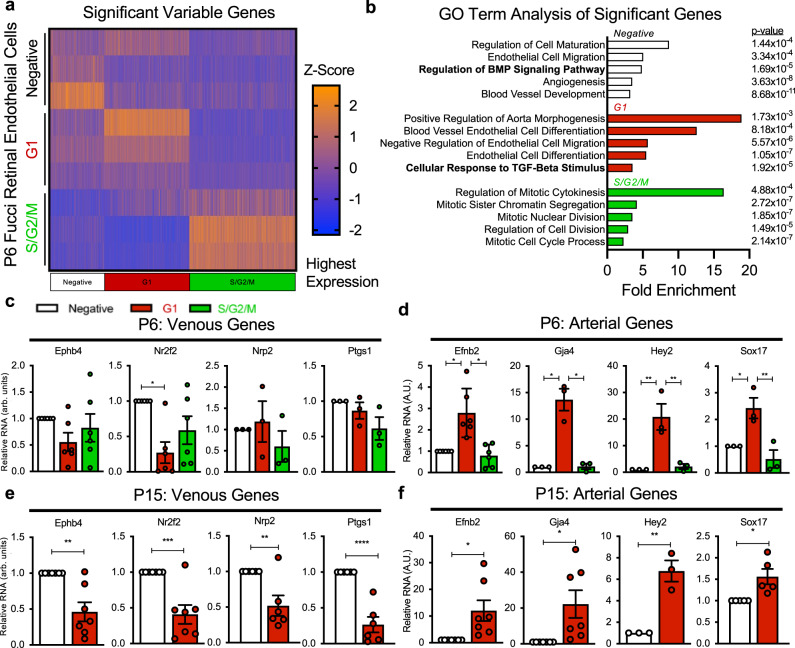
Gene expression analysis of developing retinal endothelial cells in FUCCI cell cycle states. **a** All significantly variable genes within different cell cycle states from P6 R26FUCCI2aR retinal endothelial cells from RNA sequencing, each column of heatmap shows Z-score of a single gene with enrichment in each cell cycle state highlighted by highest expression. **b** GO term enrichment analysis of genes upregulated in FUCCI-Negative state, FUCCI-Red G1 state or FUCCI-Green S/G2/M states (statistical analysis reported from GO enrichment pipeline as *p*-value). **c**–**f** Expression of venous and arterial genes in retinal endothelial cells in different cell cycle states quantified at P6 and P15 time points by qPCR (mean +/− SEM, *n* = 3–7, statistical test one-way ANOVA post hoc Tukey for c and d and two-sided *t*-test for **e** and **f**), each sample isolated from separate litters and normalized to FUCCI-Negative expression value within the same sample. Source data are provided as a Source data file. Represented *p*-values as * <  0.05, ** < 0.01, *** < 0.001, **** < 0.0001.

To further investigate the phenotypes of endothelial cells differentially expressing FUCCI reporters, we used fluorescence-activated cell sorting (FACS) to isolate P6 retinal CD31 + CD45- endothelial cells from the FUCCI reporter mice and separate them into FUCCI-Negative, -Red and -Green populations, then performed low-input RNA amplification and bulk RNA sequencing of each population (Supplementary Data 1). Analysis of these data showed that 2,691 genes are significantly variable among these populations (Fig. 2a). Gene Ontology (GO) term analysis of genes with differential expression in each population revealed that FUCCI-Negative cells are enriched for BMP signaling regulation and FUCCI-Red G1 cells are enriched for response to TGF-β stimulus. Proliferation-related GO terms were the most highly enriched in cells in S/G2/M, as expected (Fig. 2b).

Further analysis revealed that genes associated with GO Term: Mitotic Cell Cycle are highly enriched in FUCCI-Green endothelial cells, reduced in FUCCI-Negative, and further reduced in FUCCI-Red endothelial cells, suggesting a decrease in transcriptional patterns associated with cell cycle progression from FUCCI-Green to -Negative to -Red (Supplementary Fig. 2a). Consistent with these findings, analysis of the expression of known cell cycle regulators^18–21^ showed proliferation-related genes highly enriched in the FUCCI-Green endothelial cells, and genes associated with G1 state differentially expressed in FUCCI-Negative and FUCCI-Red endothelial cells (Supplementary Fig. 2b). Analysis of the expression of mitochondrial-related genes and transcription factors that promote cell proliferation (e.g., early growth response (Egr) genes)^22^ revealed enriched expression of Egr1, 2, and 3 in FUCCI-Negative endothelial cells, suggesting they are in an earlier stage of G1 compared to FUCCI-Red endothelial cells^23^ (Supplementary Fig. 2b). Enriched expression of mitochondrial-related genes in FUCCI-Red cells is consistent with a reported increase in oxidative phosphorylation in late G1 state^24^ (Supplementary Fig. 2b).

We performed additional retinal endothelial cell isolations from FUCCI reporter mice at P6 and P15, and subsequent qRT-PCR analysis, to more robustly assess differential expression of arterial and venous genes. We found that P6 endothelial cells in FUCCI-Red G1 state exhibit significantly higher expression of arterial genes and FUCCI-Negative endothelial cells exhibit increased expression of some venous genes (Fig. 2c, d and Supplementary Fig. 2c). This pattern persisted at P15, with more significant differences in arterial and venous gene expression in P15 retinal endothelial cells in FUCCI-Red G1 and FUCCI-Negative, respectively (Fig. 2e, f and Supplementary Fig. 2d).

### Single-cell analysis of retinal endothelial cell phenotype and cell cycle state

**Fig. 3:**
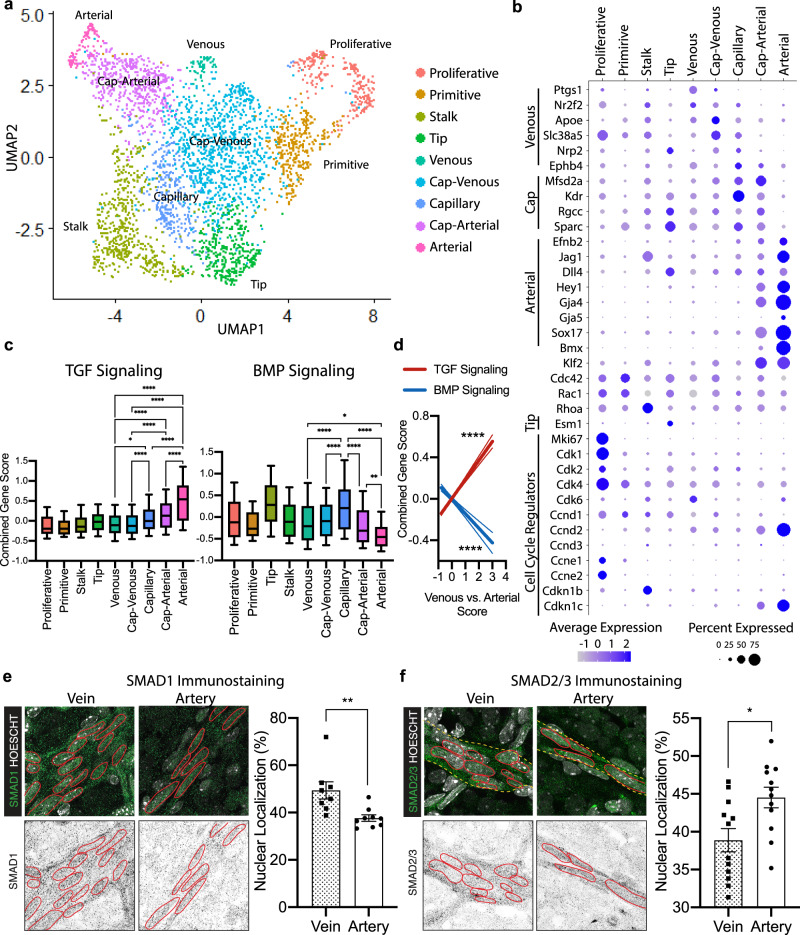
Single-cell RNA sequencing analysis of developing retinal endothelial cells. **a** UMAP dimensionality reduction plot and clustering with labeled endothelial cell populations. **b** Dot plot showing expression of genes related to venous, capillary, arterial, flow (*Klf2*), migration (*Cdc42, Rac1, Rhoa*), tip, and cell cycle regulator functions within clusters. **c** Module scoring within clusters of genes related to TGF Signaling and BMP Signaling (box = 25th−75th percentile, center = median, whiskers = 10th−90th percentile, statistical test one-way ANOVA post hoc Tukey, cells in each cluster left-to-right: *n* = 238, 312, 516, 343, 60. 955, 283, 486, 89). **d**, Linear regression analysis of each cell comparing Venous vs. Arterial Score to TGF Signaling Score or BMP Signaling Score (statistical test of simple linear regression). **e**, **f** (left panels) Representative confocal images of P6 retinal vein and artery immunostained for SMAD1 or SMAD2/3. Hoechst labels cell nuclei, outlined by dashed red lines. (right panels) Quantification of nuclear localization of SMAD1 or SMAD2/3 (mean +/− SEM, nuclear localization calculated as nuclear fluorescent intensity normalized to total intensity in the vessel × 100%, *n* = 3 retinas, 5–10 images per retina, statistical test two-sided *t*-test). Source data are provided as a Source data file. Represented *p*-values as * < 0.05, ** < 0.01, *** < 0.001, **** < 0.0001.

Endothelial cells in vascular plexi have been shown to exist in a continuum between arterial and venous phenotypes^3,25–27^. To investigate cell cycle state of endothelial cells within the continuum of arterial–venous phenotypes, we isolated endothelial cells from retinal vasculature of wild-type P6 and P15 mice and performed single-cell (sc)RNA sequencing using 10x Genomics and a drop-seq approach^28^. After filtering, we obtained 3,282 individual endothelial cells of sufficient quality for analysis (Supplementary Data 2). Seurat UMAP dimensionality reduction and clustering algorithms identified nine populations of endothelial cells within the dataset (Fig. 3a). Relative expression of venous, capillary, arterial, and cell cycle regulatory genes (along with *Esm1* and *Klf2*) were used to annotate the nine populations as Proliferative, Primitive, Stalk, Tip, Venous, Capillary (Cap)-Venous, Capillary, Cap-Arterial, and Arterial (Fig. 3b). Specifically: Proliferative cells exhibited high relative expression of *Mki67* and *Ccne1/2*; Primitive cells exhibited high expression of endothelial cell migration genes *Cdc42* and *Rac1* and no enrichment for genes typically enriched in other endothelial cell types; Tip cells showed high expression of *Esm1*; Venous and Cap-Venous showed high and medium expression of *Ptgs1*, *Nr2f2*, *Apoe*, *Slc38a5*, *Nrp2*, and *Ephb4*; Capillary showed high expression of *Mfsd2a* and *Kdr*; and Cap-Arterial and Arterial showed medium and high expression of *Efnb2*, *Jag1*, *Hey1*, *Gja4*, *Gja5, Sox17*, and *Bmx*.

In addition, cell cycle regulatory genes were differentially expressed within the populations: Proliferative and Primitive clusters showed higher expression of *Cdk1/2/4* and *Ccne1/2*, while more specified endothelial cells (Venous, Cap-Venous, Capillary, Cap-Arterial, Arterial) exhibited a gradual increase in *Ccnd2* and *Cdkn1c* across phenotypes (Fig. 3b). Endothelial cells from P6 or P15 retinas generally distributed evenly throughout the populations, except for an enrichment of the Proliferative cluster in the P6 endothelial cells and an enrichment of Cap-Venous and Cap-Arterial clusters in P15 endothelial cells (Supplementary Fig. 3a–c).

Since TGF/BMP signaling was identified in the bulk RNA sequencing results from P6 FUCCI-expressing retinal endothelial cells as differentially enriched, and these pathways have been implicated in arterial–venous specification^9,29,30^ and arterial–venous malformations^31,32^, expression of genes enriched in TGF signaling (*Smad2, Smad3*, *Tgfbr1*, *Tgfbr2*, *Acrvl1*, *Tgfb1*, *Tgfb2*, *Smad7*) or BMP signaling (*Smad1*, *Smad5*, *Bmpr2, Bmpr1a, Bmpr1b*) was used to generate module scores for each endothelial cell (Supplementary Data 3), although some genes were not highly expressed in the scRNA sequencing dataset. TGF signaling and BMP signaling scores were first assessed in each cell cluster. This analysis revealed that TGF signaling score increased, and BMP signaling score decreased, in cell clusters across the continuum from venous to arterial phenotypes (Fig. 3c). To further investigate the association between TGF/BMP signaling and the continuum from venous to arterial phenotypes, a “Venous vs. Arterial score” was calculated for each cell (Supplementary Data 3) from the relative expression of known venous or arterial genes (Supplementary Table 2). Linear regression analysis of scoring in single cells showed an association between higher Arterial Score and higher TGF Signaling score and, conversely, an association between higher Venous Scores and higher BMP Signaling Score (Fig. 3d). Immunostaining of P6 retinal vasculature for Smad1 (enriched during BMP signaling) revealed its increased nuclear localization in venous endothelial cells compared to arterial endothelial cells, indicative of greater BMP signaling in venous endothelial cells (Fig. 3e). Conversely, immunostaining for SMAD2/3 (enriched during TGF signaling) showed increased nuclear localization in arterial endothelial cells compared to venous endothelial cells (Fig. 3f).

**Fig. 4:**
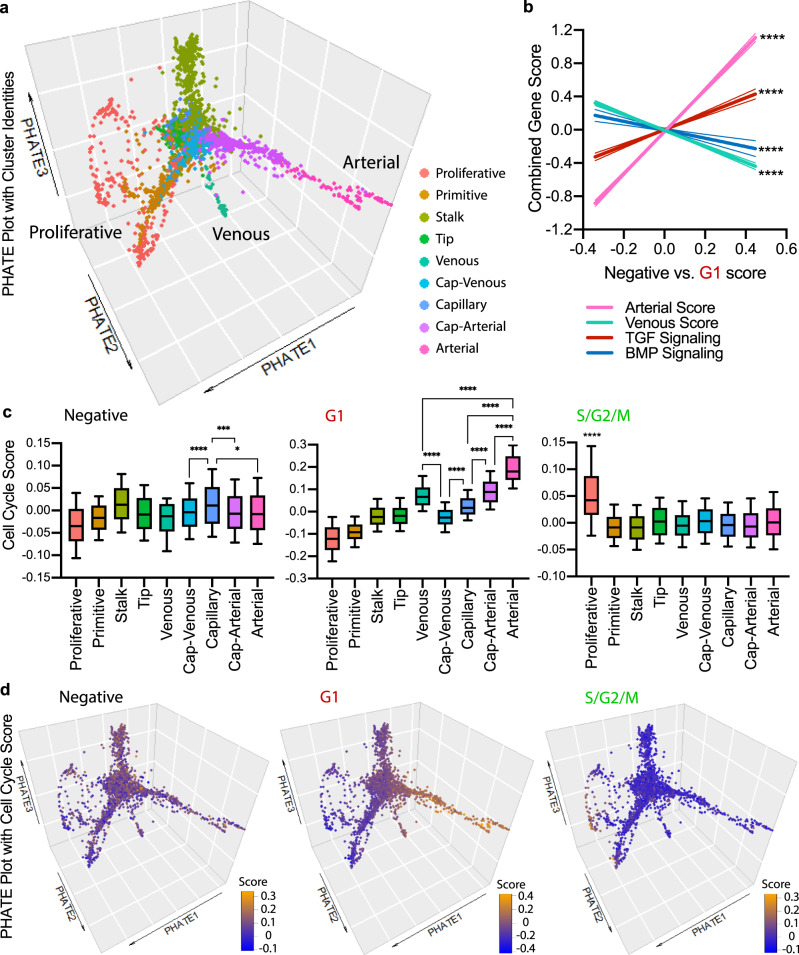
Differentiation trajectory analysis using PHATE with cell cycle scoring. **a** PHATE dimensionality reduction plot with clusters of endothelial cell populations. **b** Linear regression of relative G1 score versus Arterial, Venous, TGF Signaling, and BMP Signaling Scores in each cell. **c** Cell cycle score of FUCCI-Negative, FUCCI-Red G1 and FUCCI-Green S/G2/M within clusters determined by genes upregulated in P6 R26FUCCI2aR retinal endothelial cell bulk RNA sequencing datasets (box = 25th−75th percentile, center = median, whiskers = 10th−90th percentile, statistical test one-way ANOVA post hoc Tukey). **d** Cell cycle score FUCCI-Negative, FUCCI-Red G1 and FUCCI-Green S/G2/M of individual cells in PHATE dimensionality reduction plot. Source data are provided as a Source data file. Represented *p*-values as * < 0.05, ** < 0.01, *** < 0.001, **** < 0.0001.

To further investigate cellular associations, predicted lineage progression and cell cycle state during arterial–venous network formation, we employed PHATE dimensionality reduction analysis. This method highlights differentiation branches within scRNA sequencing datasets^33^. Three dimensions of PHATE reduction were used to accurately visualize arterial and venous phenotypes. The resulting PHATE plot suggested the different clusters progressing through differentiation pathways, including a branch containing Proliferative and Primitive cells leading into Stalk, Tip, and Capillary endothelial cells, a branch of Cap-Venous and Venous cells, and a large branch distinguishing Cap-Arterial and Arterial endothelial cells (Fig. 4a).

To assess the cell cycle state within the endothelial cell clusters, we first identified the genes enriched in endothelial cells in FUCCI-Negative, FUCCI-Red G1 and FUCCI-Green S/G2/M states in the P6 FUCCI retinal endothelial cell bulk RNA sequencing dataset. We then used cell cycle state-specific gene expression data to generate a cell cycle state “score” for each endothelial cell in the scRNA sequencing dataset (Supplementary Data 3). We first determined a relative G1 score (calculated by FUCCI-Red G1 score minus FUCCI-Negative score in each individual cell) and analyzed this score by linear regression versus Arterial, Venous, TGF Signaling, and BMP Signaling scores. This analysis showed that as FUCCI-Red G1 score increases, Arterial and TGF signaling scores increase and Venous and BMP scores decrease (Fig. 4b). In addition, we analyzed each cell cycle score in the different cell clusters, and we found, as expected, that S/G2/M scores were high in the Proliferative cell cluster. FUCCI-Negative score was slightly higher in Capillary cells, and FUCCI-Red G1 score was progressively higher from Cap-Venous to Arterial phenotypes along the venous-arterial continuum (Fig. 4c). Individual endothelial cell scores were then visualized on PHATE plot, revealing enrichment of the S/G2/M score in the proliferative branch, FUCCI-Negative score in the venous clusters, and FUCCI-Red G1 score in the arterial branch (Fig. 4d and Supplementary Movie 1).

In addition, we determined a relative G1 score (calculated by FUCCI-Red G1 score minus FUCCI-Negative score in each individual cell) in the Venous, Cap-Venous, Capillary, Cap-Arterial, and Arterial clusters. These scores were then compared to expression of venous- or arterial-enriched genes in each cell. This comparison showed that higher relative G1 score (closer to FUCCI-Red G1 state) was associated with lower expression of venous genes and higher expression of arterial genes (Supplementary Fig. 3d, e). Together, these single-cell analyses of developing retinal endothelial cells suggest that FUCCI-Negative state is associated with a venous phenotype and enriched BMP signaling, whereas FUCCI-Red G1 state is associated with an arterial phenotype and enriched TGF signaling.

### Cell cycle regulation of TGF-β/BMP signaling

Interestingly, embryonic stem cell differentiation towards specific lineages was found to be controlled by early G1 vs. late G1 cell cycle state-dependent regulation of gene expression, chromatin remodeling, and transcription factor binding^13,14^. Our in vivo observations suggest an analogous regulatory mechanism whereby different endothelial cell cycle states enable differential fate specification. Thus, to investigate the mechanistic role of endothelial cell cycle state in fate specification, we generated HUVEC expressing the Fast-FUCCI reporter [(mAG-hGem(1/110) and mKO2-hCdt1(30/120)], in which we can define cell cycle state-dependent regulation of gene and protein expression, chromatin remodeling and transcription factor binding.

To validate our model system, we first performed live-cell imaging of HUVEC-FUCCI cultured for 48 h to monitor FUCCI reporter expression during cell cycle progression. Consistent with other studies using the FUCCI reporter^10,13,34–40^, we observed that mCherry accumulates after cell division during G1 and is lost during the G1/S transition as mVenus is expressed (Supplemental Movie 2). We also performed DAPI staining to evaluate the DNA content of FUCCI cells expressing different reporters and found that cells positive for mCherry peak in the 2n population, while mVenus positive cells peak in the 4n population (Supplementary Fig. 4a).

**Fig. 5:**
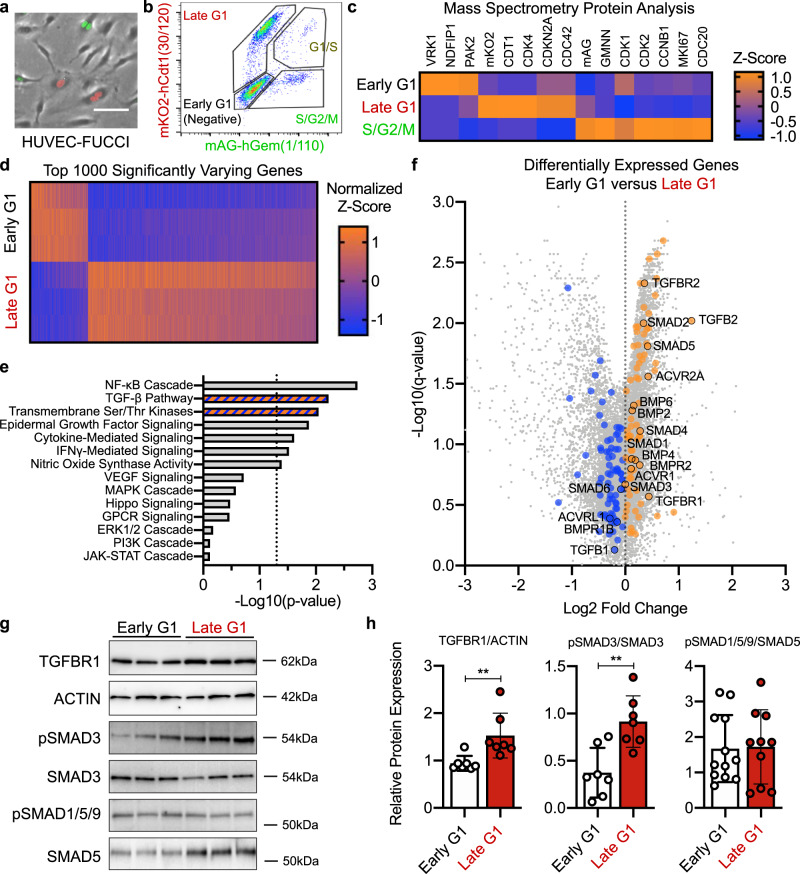
Endothelial cell cycle-dependent regulation of TGF-β/BMP pathway. **a**, **b** HUVEC-FUCCI reporter distinguishes S/G2/M, early G1 and late G1 cell cycle states, visualized by fluorescent imaging and FACS. **c** Differential expression of selected proteins within HUVEC-FUCCI cell cycle states by mass spectrometry analysis. In bulk RNA sequencing of early G1 and late G1 HUVEC-FUCCI (*n* = 3), **d** top 1000 significantly varying genes, **e** Signaling Pathway GO Term analysis (statistical analysis reported from GAGE pipeline as *p*-value), and **f** Volcano plot of fold-change against the log10(q-value) (TGF-β/BMP signaling pathway genes highlighted, upregulated in orange and downregulated in blue). **g** Western blot analysis of TGFBR1, pSMAD3 and pSMAD1/5/9 expression in early G1 and late G1 HUVEC-FUCCI. Each blot represents an independent experiment. **h** Quantification of TGFBR1, pSMAD3, and pSMAD1/5/9 in early G1 and late G1 HUVEC-FUCCI normalized to ACTIN, SMAD3, or SMAD5 respectively (mean +/− SD, *n* = 7, statistical test two-sided *t*-test). Source data are provided as a Source data file. Represented *p*-values as * < 0.05, ** < 0.01, *** < 0.001, **** < 0.0001.

We then FACS-isolated HUVEC-FUCCI into S/G2/M (green), early G1 (negative), and late G1 (red) populations (Fig. 5a, b), and isolated protein and RNA therefrom. Mass spectrometry analysis was performed on protein lysates (Supplementary Data 4), and we found cell cycle proteins to be differentially expressed, as expected, among these populations, including mAG and GEMININ that were enriched in S/G2/M cells, and mKO2 and CDT1 that were enriched in late G1 cells (Fig. 5c and Supplementary Fig. 4b). Some cell cycle proteins were not found to be differentially expressed in this analysis, but may be differentially regulated via post-translational mechanisms, such as protein phosphorylation. Bulk RNA sequencing analysis of similar populations revealed high transcriptional variation between early G1 and late G1 states (Fig. 5d and Supplementary Data 5). Of note, arterial–venous genes were not found to be differentially regulated in distinct cell cycle states in these unstimulated conditions, consistent with the idea that different cell cycle states allow for differential responses to extrinsic signals that are needed to induce arterial and venous fates.

We performed further Gene Ontology analysis on the bulk RNA sequencing dataset to determine which signaling pathways were significantly different and found that the TGF-β and transmembrane serine/threonine kinase signaling pathways are significantly variable between early G1 and late G1 (Fig. 5e). These two pathways contain many genes in the TGF-β and BMP signaling pathways that are differentially regulated in early G1 vs. late G1 (Fig. 5f). Of note, although the most enriched cell signaling pathway is NF-kB, we chose to focus on TGF/BMP signaling because similar GO terms identifying the TGF-β and BMP signaling pathways were also found in the P6 FUCCI retinal endothelial cell bulk RNA sequencing dataset, and these pathways were previously implicated in arterial–venous network formation^8,9,29,30,41^. Furthermore, via Western Blot analysis of endothelial cells in early G1 and late G1, we also found that the protein expression of some TGF-β signaling components (phospho-SMAD3 and TGFBR1) are increased in late G1 (Fig. 5g, h), suggesting that TGF-β signaling may be more active in late G1 and less active in early G1. We found no differences in noncanonical TGF-β signaling through ERK1/2 or AKT between cell cycle states or after TGF-β1/BMP4 stimulation using ligand concentrations that did not affect cell cycle state (Supplementary Fig. 5a–g). Thus, these results suggest that canonical SMAD-mediated TGF-β and BMP signaling may be differentially regulated in distinct endothelial cell cycle states, which could then enable arterial vs. venous gene induction after TGF-β or BMP stimulation, respectively. However, these RNA sequencing results do not identify directionality of the interconnected TGF-β and BMP signaling pathways in different cell cycle states, which required additional functional testing using HUVEC-FUCCI and TGF-β1/BMP4 stimulation, discussed below.

### Cell cycle regulation of arterial–venous fate

**Fig. 6:**
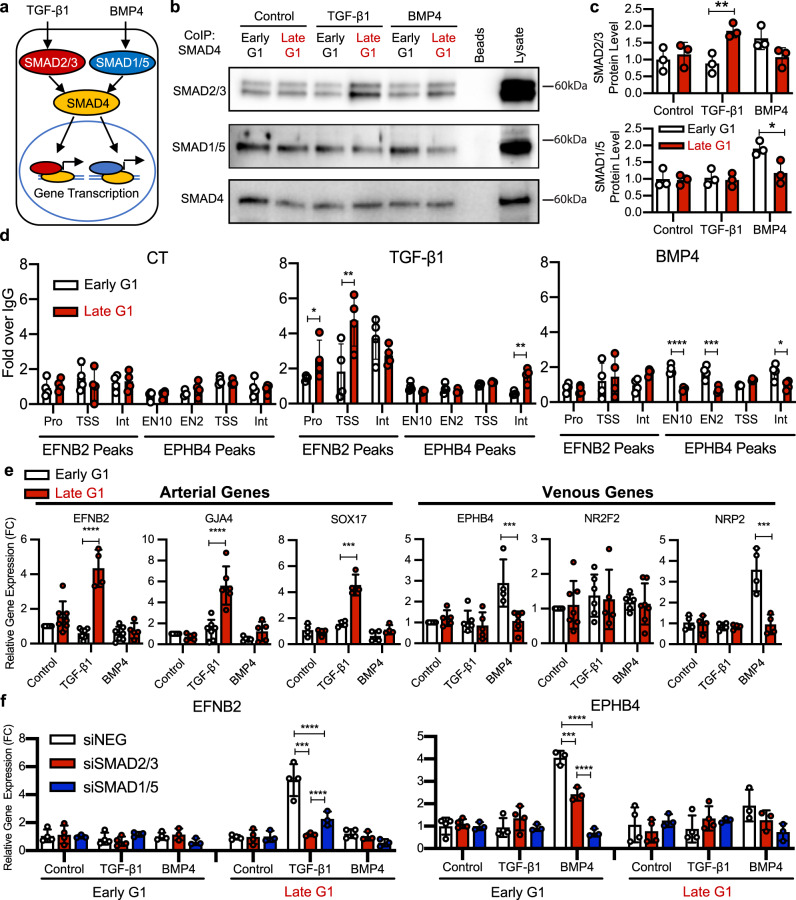
Endothelial cell cycle-dependent arterial–venous specification via TGF-β/BMP signaling. **a** Schematic of the TGF-β/BMP signaling pathway. **b**, **c** Representative Western Blot and quantification (mean +/− SD, *n* = 3) for SMAD proteins of lysates from SMAD4 co-immunoprecipitation after TGF-β1/BMP4-treated early G1 and late G1 HUVEC-FUCCI. **d** qRT-PCR analysis of DNA regions near EFNB2 and EPHB4 binding to SMAD4 complexes by chromatin-immunoprecipitation (mean +/− SD, *n* = 4). **e** TGF-β1/BMP4 induction of arterial and venous genes in early G1 and late G1 HUVEC-FUCCI (mean +/− SD, *n* = 4–6). **f** TGF-β1/BMP4 induction of EFNB2 and EPHB4 after NEG, SMAD2/3, or SMAD1/5 siRNA knockdown in early G1 and late G1 (mean +/− SD, *n* = 4). Source data are provided as a Source data file. Statistical comparison of means by two-way ANOVA post hoc Tukey, represented *p*-values as * < 0.05, ** < 0.01, *** < 0.001, **** < 0.0001.

We then examined whether TGF-β and BMP signaling are activated in endothelial cells in distinct cell cycle states. The key signaling proteins for the TGF-β/BMP signaling pathways are SMAD proteins^42^. SMAD4 serves as the intermediate co-activator, where TGF-β1 induces SMAD2/3 binding to SMAD4 and BMP4 induces SMAD1/5 binding to SMAD4^43^, and these transcription factor complexes function to promote gene expression (Fig. 6a). Using HUVEC-FUCCI, we FACS-isolated endothelial cells in early G1 and late G1 states and performed SMAD4 co-immunoprecipitation. We found that TGF-β1 (1 ng/ml for 2 h) induces greater SMAD2/3 binding to SMAD4 in late G1 state, while BMP4 (5 ng/ml for 2 h) induces greater SMAD1/5 binding to SMAD4 in early G1 state (Fig. 6b, c). Thus, BMP and TGF-β signaling appear to be differentially active in early G1 vs. late G1, respectively.

To investigate whether their downstream target genes are differentially susceptible to SMAD4 transcription factor binding in endothelial cells in distinct cell cycle states, we first performed ATAC-Sequencing to determine whether, in different cell cycle states, there is differential regulation of chromatin availability near arterial–venous genes and in enhancer regions for arterial–venous genes (Supplementary Data 6). Surprisingly, we found no significant differences in the size or location of the open chromatin regions near known arterial–venous genes, including *EFNB2* and *EPHB4*, and known enhancer regions for arterial–venous genes^9,44^ (Supplementary Fig. 6a–c). Instead, Gene Ontology analysis of the variable open chromatin peaks between early G1 and late G1 showed an enrichment of cell cycle-related genes (Supplementary Table 4). We then performed SMAD4 chromatin immunoprecipitation PCR to quantify SMAD4 transcription factor binding to their specific ATAC-seq peaks in HUVEC-FUCCI in early G1 and late G1 states, in response to TGF-β or BMP stimulation. We found that TGF-β1 (1 ng/ml for 4 h) induces more SMAD4 binding to ATAC-Seq peaks near the EFNB2 promoter and transcriptional start site in late G1 cells, while BMP4 (5 ng/ml for 4 hr) induces more SMAD4 binding to ATAC-Seq peaks near the EPHB4 enhancer and intron peaks (Fig. 6d) and the NRP2 + 26 K enhancer peak in early G1 (Supplementary Fig. 6d), with negative control chromatin regions showing no binding (Supplementary Fig. 6d). These enhancer peaks in EPHB4 (EN10 and EN2) have recently been shown to be SMAD1/5 responsive^9^. SMAD4 binding to some enhancer regions does not appear to be dependent on cell cycle, suggesting that other mechanisms may be working to control gene expression along with cell cycle state in these regions.

To further test whether BMP4 and TGF-β1 signaling promote venous vs. arterial gene expression in distinct cell cycle states, we treated HUVEC-FUCCI cells that were FACS-isolated into early G1 or late G1 states with TGF-β1 or BMP4 for 8 h (a time frame in which either treatment does not change endothelial cell cycle state, Supplementary Fig. 5c), and measured changes in mRNA expression via qPCR. We found that TGF-β1 does not induce venous gene expression in any condition, and induces arterial genes (*EFNB2*, *GJA4*, and *SOX17*) only in endothelial cells in late G1 state. Conversely, BMP4 induces venous genes *EPHB4* and *NRP2* only in endothelial cells in early G1 state (Fig. 6e). To determine if these effects were unique to HUVEC, we performed similar studies using Human Arterial Endothelial Cells expressing the FUCCI reporter (HAEC-FUCCI). We found similar results; that is, TGF-β1 induces *EFNB2* only in HAEC-FUCCI in late G1 state, and BMP4 induces *EPHB4* only in HAEC-FUCCI in early G1 state (Supplementary Fig. 7). Thus, our studies suggest that cell cycle state-mediated activation of the TGF-β and BMP signaling pathways enables differential regulation of arterial and venous genes, respectively.

Finally, we performed knockdown experiments of *SMAD* genes to determine the requirement for SMAD signaling in TGF-β1- and BMP4-induced arterial–venous gene expression. Transfection of siRNA yielded >80% knockdown of targeted *SMAD* genes. We found that siRNA-mediated knockdown of *SMAD2/3* prevents TGF-β1-induced *EFNB2* gene induction in late G1, and knockdown of *SMAD1/5* prevents BMP4-induced *EPHB4* gene induction in early G1 (Fig. 6f). Also, although we observed lower TGF-β1-induced *EFNB2* expression in the *siSMAD1/5* treated cells and lower BMP4-induced *EPHB4* in the *siSMAD2/3* treated cells, these results are not unexpected as these signaling pathways are highly interdependent, with crosstalk among signaling components^45^. Nonetheless, our results suggest that late G1 state is required for TGF-β1-mediated arterial gene induction through SMAD2/3, and early G1 state is required for BMP4-mediated venous gene induction through SMAD1/5 in endothelial cells.

### Prevention of arterial–venous fate defects

To investigate the role of endothelial cell cycle state in arterial–venous specification in vivo, we tested whether pharmacological manipulation of cell cycle state could prevent arterial–venous specification defects that have been associated with endothelial cell hyperproliferation. We used Connexin (Cx)37-deficient mice (Cx37-KO) in which we previously found endothelial cell hyperproliferation, downregulated cell cycle inhibitor p27 (Cdkn1b), disrupted retinal vascular plexus remodeling and impaired arterial blood vessel maturation^2^. p27 induces G1 arrest in cells by interacting with and inhibiting the Cyclin D-CDK4 and Cyclin E-CDK2 complexes^46,47^, which normally function to promote G1-to-S transition^48^.

**Fig. 7:**
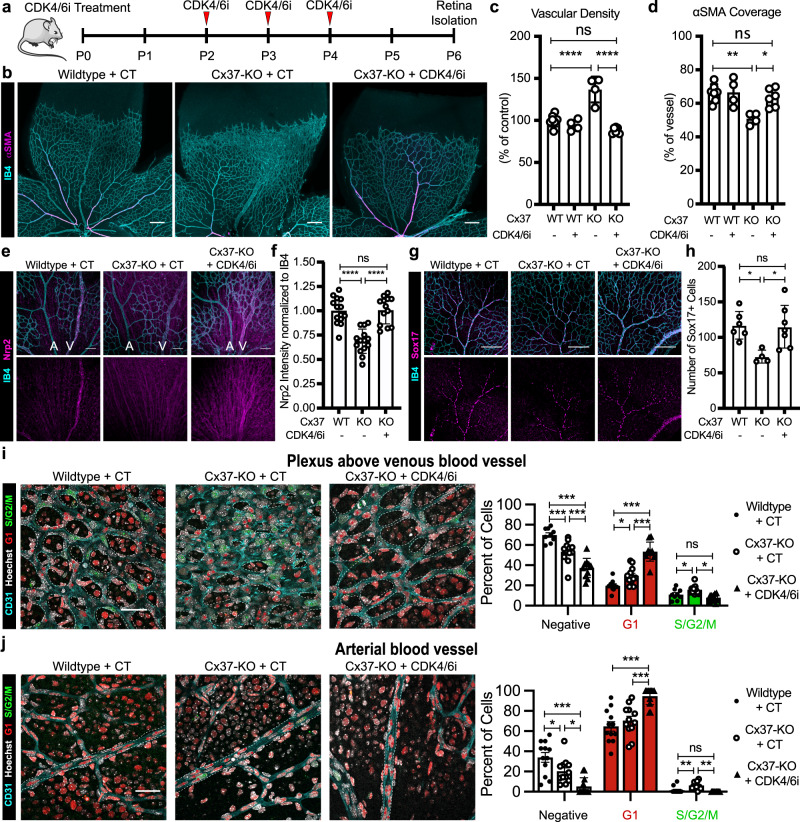
Rescue of arterial–venous development defects with pharmacological CDK4/6 inhibition. **a** CDK4/6i treatment timeline. **b** Representative confocal images of P6 retinas from WT, Cx37-KO, and Cx37-KO + CDK4/6i treated immunostained with IB4 and anti-αSMA (scale bars = 200 μm), quantified for: **c** vascular density (mean +/− SD), **d** αSMA vascular coverage (mean +/− SD). **e** P6 retinas from WT, Cx37-KO, and Cx37-KO + CDK4/6i treated mice immunostained with anti-Nrp2 and IB4 (scale bar = 150 μm). **f** Quantification of Nrp2 intensity in retinal veins and arteries normalized to IB4 intensity (mean +/− SD). **g** P6 retinas from WT, Cx37-KO, and Cx37-KO + CDK4/6i treated mice immunostained with anti-Sox17 and IB4 (scale bar = 150 μm). **h** Quantification of Sox17+ cells in retinal arterial branches (mean +/− SD). **i**, **j** (left panels) Representative confocal images of P6 retina from R26p-FUCCI2, R26p-FUCCI2 + Cx37-KO, and R26p-FUCCI2 + Cx37-KO + CDK4/6i treatment immunostained with IB4 and Hoechst (scale bars = 50 μm). Vessels outlined in dotted white lines, cell cycle state highlighted with colored stars. (right panels) Quantifications of cell cycle states in plexi above venous blood vessels and arterial blood vessels (mean +/− SEM). Source data are provided as a Source Data file. Statistical comparison of means by one-way ANOVA post hoc Tukey (**c**, **d**, **f**, **h**) or two-way ANOVA post hoc Tukey (**i**, **j**), represented *p*-values as * < 0.05, ** < 0.01, *** < 0.001, **** < 0.0001.

Cell cycle state can be regulated through pharmacological inhibition of CDK proteins. Specifically, we previously found that CDK4/6i (Palbociclib, PD-0332991) reduces active cycling (S/G2/M) and promotes growth arrest^2^. Therefore, we treated Cx37-KO mice with CDK4/6i during early retinal vascular development (P2-4) and assessed its impact on vascular remodeling and arterial–venous specification at P6 (Fig. 7a). We found that the vascular hyper-density and arterial maturation defects (indicated by reduced αSMA coverage) observed in P6 retinas of Cx37-KO mice are both prevented with CDK4/6i treatment (Fig. 7b–d). In addition, we found that endothelial cells in Cx37-KO retinal vasculature exhibited reduced expression of Nrp2 (venous identity^49,50^), and reduced expression of Sox17 in arterial branches (early arterial identity^50,51^). Treatment of Cx37-KO mice with CDK4/6i prevented these defects in arterial–venous endothelial cell development (Fig. 7e–h) and did not significantly affect Cx37-KO mouse growth/weight (Supplementary Fig. 8a). These results suggest that CDK4/6i treatment prevented the arterial–venous specification defects observed in the Cx37-KO retinal vasculature.

We then investigated developing retinal endothelial cell cycle state in Cx37-KO mice by crossing Cx37^−/−^ mice^52^ with R26p-FUCCI2 mice^53^ that globally express the FUCCI construct to generate Cx37^−/−^;R26p-FUCCI2 mice. We compared Cx37-KO mice to wild-type controls, as well as the effects of CDK4/6i treatment on endothelial cell cycle state during vascular remodeling. Firstly, we found that, in Cx37-KO mice compared to wild-type controls, a higher proportion of endothelial cells in arterial branches and plexi above the venous branches are actively cycling (S/G2/M), and a lower proportion of endothelial cells in the venous branch and the plexi above arterial and venous blood vessels are FUCCI-Negative (Fig. 7i, j and Supplementary Fig. 8b, c). Interestingly, a higher proportion of endothelial cells in the plexi above the venous and arterial branches are also in FUCCI-Red G1.

When Cx37^−/−^;R26p-FUCCI2 mice were treated with the CDK4/6i, they exhibited an increased proportion of endothelial cells in the remodeling plexi above the venous blood vessels in FUCCI-Red G1 state and a reduced proportion in FUCCI-Negative and S/G2/M states (Fig. 7i). In the arterial blood vessels, we also found significantly more endothelial cells in FUCCI-Red G1 after CDK4/6i treatment and fewer endothelial cells in S/G2/M and FUCCI-Negative states (Fig. 7j). We did not observe large changes in the cell cycle state of endothelial cells within venous branches themselves or the plexi above the arterial blood vessels (Supplementary Fig. 8b, c). We further confirmed that CDK4/6i treatment results in reduced endothelial cell proliferation assessed by EdU incorporation (Supplementary Fig. 8d). Although we did not observe a reduction in FUCCI-Red G1 state between wild-type and Cx37-KO retinal endothelial cells, CDK4/6i treatment of Cx37-KO mice resulted in a greater proportion of endothelial cells in FUCCI-Red G1 within arteries and the plexi above veins (Fig. 7i, j). Thus, we found that the CDK4/6i-induced increased retinal endothelial cells in FUCCI-Red G1 and reduction in S/G2/M in the Cx37-KO mice is sufficient to prevent their defects in arterial–venous development and vascular remodeling.

## Discussion

These studies reveal that arterial and venous endothelial cells reside in different cell cycle states during development and in adulthood. Endothelial cells in venous branches are predominantly FUCCI-Negative and endothelial cells in arterial branches predominantly express the FUCCI-Red reporter which, in the FUCCI2a mice that we employ, is expressed by cells in G1 and G0^10,12^. The FUCCI-Negative cells are thought to be in an earlier stage of G1^10^, which is supported by other in vivo studies^54–56^, and our transcriptomic analyses of developing FUCCI-expressing retinal endothelial cells. However, in other studies, the FUCCI-negative state is interpreted to represent “early G1/G0” in vivo^57,58^; thus, the FUCCI reporter read-out may be context-dependent and/or have technical limitations. Nonetheless, our in vivo studies of retinal vascular development demonstrate associations between FUCCI-Negative retinal endothelial cells, venous phenotype, and BMP signaling, as well as FUCCI-Red retinal endothelial cells, arterial phenotype, and TGF-β signaling. These effects may be mediated via the activation of distinct signaling pathways that differentially regulate endothelial cell cycle state. For example, previous studies showed arterial shear stress activates Notch signaling to promote p27-mediated G1 arrest that enables arterial gene expression^2^. However, given that endothelial cell growth arrest can be induced by other microenvironmental factors^4^, there are likely multiple interactive pathways that regulate endothelial cell cycle state in vivo.

Our mechanistic studies, performed in a validated HUVEC-FUCCI model, are the first to identify a molecular link between cell cycle state and propensity for endothelial cell fate, and they provide a framework that integrates other observations in the field. For example, TGF-β and BMP signaling have been implicated in the regulation of arterial–venous gene expression^9,29^ and their dysregulation can lead to arterial–venous malformations^31,32^; however, it was not clear how these pathways could be coordinated in close proximity to establish an arterial–venous network. Our findings show that the early G1 state will license endothelial cells to be permissive for BMP signaling and thereby allow venous specification. In contrast, late G1 state is more permissive for TGF-β signaling that promotes arterial specification. Thus, these studies provide a paradigm of arterial vs. venous phenotypic specialization that involves cell cycle-mediated control of distinct signaling pathways that serve to enable arterial vs. venous patterns of gene expression.

Our findings are also consistent with recent publications suggesting that endothelial cells with a Tip cell phenotype can acquire an arterial phenotype and contribute to arterial vessel development^16,17^. Analysis of the FUCCI-expressing P6 retinal vasculature revealed no Tip cells in S/G2/M phases of cell cycle, and a higher percentage in FUCCI-Red G1 compared to surrounding cells. Our mechanistic results suggest that the Tip cells will be more permissive for signals that promote arterial specification. The fact that Tip cell growth arrest was found to be mediated via Notch signaling^59^ is also consistent with our previous studies showing that shear stress-activated Notch signaling promotes arterial specification through p27-mediated growth arrest^2^.

Other recent studies have highlighted the importance of cell cycle regulation in endothelial cell differentiation. Coronary artery development is impaired when cell cycle control is disrupted by overexpression of Nr2f2 to promote proliferation in the progenitor endothelial cells coming from the sinus venosus^3^. In addition, we previously found that p27-mediated cell cycle arrest is also required for endothelial cells to undergo hemogenic specification during definitive hematopoiesis^60^, and retinoic acid promotes early G1 arrest in human endothelial cells in vitro to enable hemogenic specification^61^. Thus, regulation of cell cycle state may be required to enable the phenotypic specialization of all endothelial cell subtypes.

Our unbiased sequencing studies enabled a broader view of the relationship between endothelial cell cycle state and phenotype. However, the FUCCI reporter limits our analysis to discrete cell cycle states, but endothelial cell phenotype during development and in the adult vasculature has been shown to exist in a continuum between arterial and venous phenotypes^3,25–27^. Furthermore, endothelial cells within the same blood vessel can show remarkable heterogeneity both in phenotype and in the response to injury^25,62–64^. This endothelial cell heterogeneity may extend to cell cycle status and explain why we did not find complete distinction of FUCCI cell cycle states in arteries vs. veins. In support of this idea, analysis of the scRNA sequencing dataset, based on “cell cycle scores” obtained from bulk RNA sequencing of endothelial cells expressing different FUCCI reporters, showed trends in cell cycle states associated with phenotypic changes.

Interestingly, the scRNA sequencing results showed that known venous genes are not as highly enriched compared to arterial genes during retinal vascular development. This may highlight a difference in timing between arterial and venous specification and maturation. In addition, the FUCCI-Negative scoring was not as well defined as the FUCCI-Red G1 scoring. However, investigating cell cycle scoring and arterial–venous scoring at the single-cell level showed significant associations. That is, endothelial cells with increasing FUCCI-Red G1 scores exhibited reduced venous gene expression and greater arterial gene expression. These results suggest that endothelial cell phenotypes within the arterial–venous continuum also exhibit a continuum of cell cycle states.

The distinction of cell cycle states between the endothelial cells of veins and arteries is maintained into adulthood. Indeed, arterial–venous malformations can be associated with underlying endothelial cell hyperproliferation that may contribute to disruptions in arterial–venous identity^30,49,65,66^. This is of particular interest given that pharmacological cell cycle arrest was found to be sufficient to prevent endothelial cell hyperproliferation and defects in arterial–venous specification in an animal model of dysregulated vascular network formation. These experiments employed the CDK4/6 inhibitor Palbociclib that is FDA approved for the treatment of HR-positive, HER2-negative metastatic breast cancer^67,68^, and is currently being investigated for other forms of cancer^69–71^. Thus, Palbociclib or similar drugs may be beneficial for the treatment of vascular diseases, such as arteriovenous and cerebral cavernous malformations, and they may also have utility for the control of arterial–venous identity during the creation of arteriovenous fistulas, coronary artery bypass grafting, and vascular procedures.

**Fig. 8:**
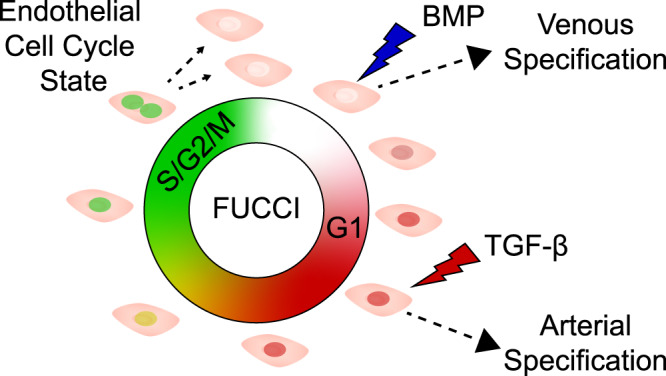
Cell cycle state determines propensity for arterial–venous specification hypothesis. Early G1 state allows for greater induction of venous specification by BMP signaling, while late G1 state allows for greater induction of arterial specification by TGF-β signaling.

In summary, our mechanistic studies reveal that endothelial cell cycle state determines propensity for arterial–venous specification (Fig. 8). Early G1 state allows for enhanced induction of venous specification by BMP signaling, and late G1 state enables TGF-β-induced arterial specification. Future studies that address intermediate endothelial cell phenotypes and further investigate mechanisms of cell cycle-dependent regulation of signaling pathways will enable deeper understanding of vascular development and vascular pathologies.

## Methods

### Mouse strains

All animal procedures were approved by the Institute for Animal Care and Use Committees at Yale University and the University of Virginia. Animal housing was controlled by Yale and University of Virginia Vivarium Staff, maintaining a 14 h light to 10 h dark cycle, an ambient temperature of 21.5 °C (acceptable range 20–26 °C), and an ambient humidity 40% (acceptable range 30–70%). In this study, R26FUCCI2aR mice^10^ were bred with Cdh5-CreER^T2^ mice^11^ to generate endothelial-specific FUCCI expression after tamoxifen injection. Tamoxifen (Sigma Cat# T5648) was resuspended in 10% EtOH and 90% Corn oil (Sigma Cat# C8267) at 4 mg/mL, and 25 μL was injected per pup. In addition, Gja4−/− (Cx37-KO) mice^52^ were bred with R26p-FUCCI2 mice^53^ to generate FUCCI expression in Cx37-KO mice. All experiments used equal numbers of male and female mice. All mice were on a C57BL6 background.

### Tissue isolation, staining, and quantification

Retinas were isolated from mice, immunostained, and imaged as previously described^2^. Briefly, retinas were carefully dissected from PFA-fixed eyes, immunostained with given antibodies, and imaged using fluorescence confocal microscopy. Embryos were isolated at E9.5, E10.5, and E11.5, femoral arteries and veins were isolated from adult 8-week-old adult mice, and all tissues were fixed by 4% PFA for 2 h, then sectioned by cryosectioning and immunostained similar to retinal staining. All quantification of tissue staining was performed on at least three independent replicate experiments including at least three separate litters. All antibodies are listed in Table S1. For retina imaging, isolated retinas were immunostained for IB4, CD31, Erg1/2/3, or αSMA, with fluorescent secondary antibodies. Stained retinas were imaged by confocal microscopy (Leica SP8). Endothelial cells along arterial branches and venous branches were quantified by Erg1/2/3 expression in the nuclei, and cell cycle was determined by FUCCI expression (FUCCI-Negative by mCherry^-^mVenus^−^; FUCCI-Red G1 by mCherry^+^mVenus^−^; FUCCI-Green S/G2/M by mCherry^−^mVenus^+^). Endothelial cells were isolated through a previously described and published method optimized for purification and viability^72^. Briefly, dissected retinas were digested with Collagenase Type II (1.0 mg/mL, Gibco Cat# 17101015) in DMEM (Gibco Cat# 21013024) and 10% FBS (Gibco Cat# 26140079) for 20 min, washed, stained for anti-CD31 and -CD45 in staining buffer (HBSS with 10% FBS, 20 mM HEPES, 1 mg/mL D-Glucose), then resuspended in FACS buffer (PBS with 1% FBS). FUCCI cell populations were isolated by FACS through a CD31^+^CD45^−^ gating strategy, then by mCherry/mVenus to determine cell cycle. Cells were sorted into RNA lysis buffer, and RNA was purified with RNeasy Micro Kit (Qiagen Cat# 74034). FACS was performed with a BD FACSAria at either the Yale Flow Cytometry Core or the University of Virginia Flow Cytometry Core or a BD FACSMelody. Flow cytometry data analysis was performed with FlowJo, Version 10.6.1.

### Retinal endothelial cell bulk RNA sequencing

Endothelial cells from retinal tissue of P6 R26FUCCI2aR mice of three separate litters were isolated as described above. Single-cell suspensions of endothelial cells were separated by FACS into FUCCI-Green S/G2/M, FUCCI-Negative, and FUCCI-Red G1 populations by gating on mCherry and mVenus channels. Low cell isolation numbers required cDNA amplification for RNA sequencing. RNA preparation and cDNA amplification for Illumina next-generation sequencing was performed with the SMART-Seq v4 Ultra Low Input RNA Kit for Sequencing (Takara Bio USA, Inc.). Samples were then transferred to the University of Virginia Genome Analysis and Technology Core, which performed cDNA library preparation with the DNA Prep, (M) Tagmentation kit (Illumina Cat #20018704) and sequenced samples with an Illumina NextSeq 500 High Output at 2 × 75 paired-end reads. Three paired biological replicates were sequenced for a total of nine samples. Raw read data was quality-control checked (FastQC, v0.11.9), aligned to mouse genome GRCm38 using Kallisto v0.46.2^73^, and analyzed for total and differential gene expression using Sleuth v0.30.0^74^. Gene Ontology analysis was performed with GAGE v2.40.2^75^.

### Quantitative RT-PCR

Purified RNA was converted to cDNA using the High-Capacity cDNA Reverse Transcription Kit (ThermoFisher Cat# 4368814) and quantified by Power SYBR Green PCR Master Mix (ThermoFisher Cat# 4368577) via qRT-PCR (Applied Biosystems QuantStudio 6). Gene-specific primers are listed in Supplementary Table 3. Relative quantification was determined by the delta-delta-CT method. Quantification was performed in at least three biological replicates for each experiment.

### Single-cell RNA sequencing and bioinformatic analysis of retinal endothelial cells

Endothelial cells from P6 and P15 wild-type mice were isolated as described above, and single-cell suspensions of endothelial cells were selected through FACS of CD31 + CD45− immunostaining. Single-cell RNA sequencing was then performed by the Yale Center for Genomic Analysis, which used around 12,000 cells per sample through the 10x Genomics Drop-Seq platform to generate single-cell barcoded cDNA libraries. Next-generation sequencing of single-cell barcoded cDNA libraries was performed in collaboration with WuXi NextCODE (Boston, MA and Shanghai, China). Single-cell RNA sequencing datasets were analyzed by Seurat v3.0 to filter out cells by high mitochondrial gene expression, low or high gene numbers, and contaminating cell types (Hbb.bt, Neurod1, Cd63). P6 and P15 datasets were integrated by IntegrateData function using SCT normalization. Principal Components that accounted for 90% data variability were used (first 13) to determine UMAP dimensionality reduction, and FindClusters function with resolution of 0.5 was used to determine clusters. The AddModuleScore function was used with specific genes found in the dataset to generate scores: TGF Signaling (Smad3, Tgfbr1, Tgfbr2, Smad6, Acvrl1, Tgfb1, Tgfb2), BMP Signaling (Smad1, Smad5, Bmpr2) and cell cycle scoring with genes enriched in P6 FUCCI retinal endothelial cell bulk RNA sequencing states. In addition, single-cell RNA sequencing datasets were analyzed by PHATE v0.3.0 analysis^33^. The phate function with 3 dimensions was used on all integrated gene expression data obtained from Seurat analysis. The MAGIC v3.0.0 imputation algorithm^76^ was used with parameters *t* = 4 on all expressed genes to account for gene dropout in PHATE analysis. Arterial Score and Venous Score were determined by average relative expression of arterial genes or venous genes after imputation (genes in Supplementary Table 2), and Arterial vs. Venous Score was calculated in each cell by Arterial Score minus Venous Score. Three-dimensional PHATE plots were generated in R using the plot3D function.

### Protein analysis by mass spectrometry

Harvested cells were pelleted and frozen at −80 °C. Each sample was prepared according to protocol using the EasyPep Mini MS Sample Prep Kit (ThermoFisher, A40006) with Halt Phosphatase Inhibitor Cocktail (ThermoFisher, 78420) added to the lysis solution. Preparation of 50 μg of protein per sample was performed according to kit protocol, including reduction and alkylation of cysteines. 10 μg of Pierce Trypsin/Lys-C protease mix was added to each sample and incubated with shaking at 37 °C for 1.5 h, then samples were desalted using C18 spin columns, eluted, and dried via speed vacuum centrifugation. Samples were reconstituted in 50 μL of 0.1% formic acid for a final concentration of 1 μg/μL. The resulting peptides were analyzed by nanoLC-MS/MS using a Dionex Ultimate 3000 (Thermo Fisher Scientific, Bremen, Germany) coupled to an Orbitrap Eclipse Tribrid mass spectrometer (Thermo Fisher Scientific, Bremen, Germany). 5 μL of each sample was loaded onto an Acclaim PepMap 100 column (75 μm × 25 cm, 3 μm C18) equilibrated in 94% solvent A (0.1% formic acid in water) and 4% solvent B (80% acetonitrile in 0.1% formic acid). The peptides were eluted at 300 nL/min using the following gradient: 4% B from 0 to 5 min, 4–10% B from 5 to 10 min, 10–35% B from 10 to 100 min, 35–55% B from 100 to 120 min and 55–90% B from 120 to 121 min. The samples were run in triplicate with three blank gradients in between each sample. The Orbitrap Eclipse was operated in positive ion mode with 1.9 kV at the spray source, RF lens at 30% and data-dependent MS/MS acquisition with XCalibur software. Positive ion Full MS scans were acquired in the Orbitrap from 375 to 1500 *m*/*z* with 120,000 resolution. Data-dependent selection of precursor ions was performed in Cycle Time mode, with 3 s in between Master Scans, using an intensity threshold of 1e4 ion counts and applying dynamic exclusion (*n* = 2 scans within 30 seconds for an exclusion duration of 60 s and +/−10 ppm mass tolerance). Monoisotopic peak determination was applied and charge states 2–7 were included for HCD/ETD toggle scans (quadrupole isolation mode; 1.6 *m*/*z* isolation window). The resulting fragments were detected in the ion trap with Rapid scan rate setting and standard AGC target. Proteome Discoverer (PD) software (version 2.4.0.305 Thermo Fisher Scientific, city) was used to perform database searches of raw files. Data files were searched against the gencode.v36.pc_translations protein database with sequences for mKO2 and mAG proteins manually added to the fasta file. The Sequest HT search algorithm was used, with precursor and fragment mass tolerances set to 10ppm and 0.6 Da, respectively. The enzyme specificity was set to trypsin (full) with up to two missed cleavages allowed. Carbamidomethyl on C was used as a static modification and oxidation on M and phosphorylation on S,T,Y were considered as variable modifications on amino acids. Strict parsimony settings were used for protein grouping, false discovery rates for peptide spectrum match and protein were set to 0.01 and determined using the Percolator node of PD with decoy database search. The Minora Feature Detector node and Precursor Ions Quantifier node were used to perform relative quantitation with default settings. Only high confidence peptide spectrum matches were considered with the Minora Feature Detector. For Quan Rollup and Hypothesis Testing, summed abundances were used for protein abundance calculations, using a pairwise ratio and ANOVA (individual proteins) for hypothesis testing.

### Generation of HUVEC-FUCCI and HAEC-FUCCI

Primary Human Umbilical Vein Endothelial Cells (HUVEC) were obtained from the Yale Vascular Biology and Therapeutics Core. Human Aortic Endothelial Cells (HAEC) were purchased (Lonza, Cat# CC-2535). Endothelial cells were passaged in Endothelial Cell Growth Medium (PromoCell Cat# C-22010) and experiments were performed in EGM-2 (Lonza Cat# CC-3162). Experiments used cells between passage 4 and 8. HUVEC and HAEC were infected with lentivirus generated with HEK293T cells infected with the Fast-FUCCI plasmid^15^; pBOB-EF1-FastFUCCI-Puro was a gift from Kevin Brindle & Duncan Jodrell (Addgene plasmid # 86849). Cells were selected and passaged in Puromycin (1 μg/mL, Sigma P9620). Live-cell imaging of HUVEC-FUCCI was performed over 48 h by fluorescent and brightfield microscopy (Leica DMi8 inverted research microscope with Lumencor Spectra X LED light source and Thunder deconvolution software) while cells were grown in an environmental control unit maintaining 37 °C, 90% humidity and 5% CO_2_ (Okolab).

### FACS of HUVEC-FUCCI

Sorting of HUVEC-FUCCI into cell cycle states was performed by lifting subconfluent cells from cell culture with Accutase (Sigma Cat# A6964) or 0.25% Trypsin-EDTA (Gibco Cat# 25200056), washing cells and resuspending in FACS buffer. Fluorescent levels of mCherry and mVenus were used to determine cell cycle state. Cells were either sorted by FACS with a BD FACSAria or BD FACSMelody, or analyzed by flow cytometry with a BD LSRII (BD Biosciences). Quantification performed on at least *n* = 3 biological replicates.

### HUVEC-FUCCI bulk RNA sequencing and data analysis

RNA from HUVEC-FUCCI sorted into different cell cycle states (*n* = 3 biological replicates) was purified and submitted for next-generation transcriptome sequencing to the Yale Center for Genomic Analysis (Illumina HiSeq4000). Raw read data was quality-control checked (FastQC, Version 0.11.9), aligned to human genome hg38 using Kallisto v0.46.2^73^, and analyzed for total and differential gene expression using Sleuth v0.30.0^74^. Gene Ontology analysis was performed with GAGE v2.40.2^75^.

### Western blot analysis

Protein was isolated from cells using RIPA Buffer (Sigma Cat# R0278) or sorted directly into Laemmli Buffer (BioRad Cat# 1610747). Western blot analysis was performed using the Criterion Vertical Electrophoresis Cell (BioRad Cat# 1656020) with 4–15% Criterion Tris-HCl Protein Gels (BioRad Cat# 3450028) and imaged with the Azure Biosystems c300. Western blots were quantified by ImageJ densitometry analysis.

### TGF-β1/BMP4 ligand induction

Subconfluent, HUVEC-FUCCI were lifted and sorted into early G1 and late G1 cell cycle states, then seeded at 5 × 10^4^ cells per well into 6-well plates. Cells were left to attach to the plate for 1 h in a 37 °C, 5% CO_2_ incubator, then media was changed with new media supplemented with control, TGF-β1 (1 ng/mL, R&D Systems Cat# 240-B) or BMP4 (5 ng/mL, R&D Systems Cat# 314-BP). These concentrations were optimized for gene induction without affecting cell cycle state (Supplemental Fig. 5c). For co-immunoprecipitation experiments, cells were incubated in ligands for 2 h, then SMAD4 complexes were isolated using the Pierce Co-Immunoprecipitation Kit (ThermoFisher Cat# 26149) per manufacturer’s instructions. For chromatin immunoprecipitation experiments, cells were incubated in ligands for 4 h, then SMAD4-DNA complexes were isolated using the High-Sensitivity ChIP Kit (AbCam Cat# ab185913), per manufacturer’s instructions. For gene induction experiments, cells were incubated in ligands for 8 h, then RNA lysate was collected and qRT-PCR was performed.

### HUVEC-FUCCI ATAC-sequencing and data analysis

Subconfluent, HUVEC-FUCCI were lifted and sorted into early G1 and late G1 cell cycle states, then immediately collected for ATAC-sequencing analysis (*n* = 4 biological replicates). Library preparation was performed as previously described^77^. Briefly, cells were pelleted by 13,000 rpm for 1.5 min in a tabletop centrifuge, cells were resuspended in 50 μL Transposase Reaction Mix (25 μL 2x TD Buffer, 2.5 μL Transposase Enzyme from Illumina Nextera Cat# FC121-1030, 22 μL nuclease-free water, 0.5 μL 0.1% digitonin) and incubated for 30 min at 37 °C, then DNA was purified using a QIAGEN MinElute PCR Purification Kit (Cat# 28004), amplified using KAPA HiFi HotStart ReadyMix PCR kit, and sequenced tagged primers according to the published protocol, then amplified DNA was purified using AMPure Beads (Beckman Coulter Cat# A63880). Sequencing was performed at the Yale Center for Genomic Analysis (Illumina HiSeq4000). Raw read data was quality controlled with FastQC v0.11.9 (Babraham Bioinformatics), filtered and trimmed with Trimmomatic v0.33^78^, then peaks were called with MACS2 v2.2.7.1^79^, and differential peak analysis was performed with HOMER v4.11 (UCSD).

### Transfection of siRNA

HUVEC-FUCCI were transfected with ThermoFisher Silencer Select siRNA targeting SMAD1 (Cat# s8394), SMAD2 (Cat# s8397), SMAD3 (Cat# s8400), SMAD5 (Cat# s8406), or Negative Control (Cat# 4390843). RNAiMAX Lipofectamine (ThermoFisher Cat# 13778075) was used to package and transfect siRNA into HUVEC-FUCCI, per manufacturer’s instructions. After 48 h, HUVEC-FUCCI were lifted and sorted by FACS into early G1 or late G1, then induced with ligand, as described above.

### Cdk4/6 inhibitor administration in mice

Cdk4/6 inhibition in mice was performed by resuspending Palbociclib (Sigma-Aldrich Cat# PZ0383) in 50 mM Sodium lactate (Sigma Cat# L7022) at 12 mg/mL, then administered to pups at P3, P4, and P5 by oral gavage. Retinas were isolated at P6. EdU incorporation assay was performed using the Click-It EdU Cell Proliferation Kit (ThermoFisher Cat# C10340) per manufacturer’s instructions, with EdU injection into mice 5 h before euthanasia at P6.

### Statistics and reproducibility

Unless otherwise indicated, statistical analysis was performed using either a standard two-tail Student’s *t*-test or a two-way ANOVA test followed by a Tukey’s multiple comparison corrected post hoc test. All statistical analysis of mass spectrometry protein analysis, RNA sequencing and ATAC sequencing datasets was performed through computational analysis packages, which incorporate statistical corrections for large datasets. All experiments were repeated independently with similar results at least three times. Specifically, retinal vascular imaging from Cdh5CreER^T2^;R26FUCCI2aR mice at P6 and P15 was repeated in six litters with similar results, as well as repeated in the R26p-FUCCI2 mice with similar results. Imaging of HUVEC-FUCCI was repeated three times, as well as analyzed with time-lapse imaging. Retinal vascular imaging from Cx37-KO mice with CDK4/6i treatment was repeated four times.

### Reporting summary

Further information on research design is available in the Nature Research Reporting Summary linked to this article.

## Supplementary information

Supplementary Information

Description of Additional Supplementary Files

Supplementary Movie 1

Supplementary Movie 2

Supplementary Data 1

Supplementary Data 2

Supplementary Data 3

Supplementary Data 4

Supplementary Data 5

Supplementary Data 6

Reporting Summary

## Data Availability

Source data are provided as a Source data file. The bulk RNA sequencing datasets generated in this study have been deposited in the NCBI GEO database under accession code GSE211658. The single-cell RNA sequencing dataset of P6 and P15 retinal endothelial cells generated in this study has been deposited in the NCBI GEO database under accession code GSE169039. Proteomics datasets generated in this study have been deposited in the PRIDE database under accession code PXD036326. The bulk ATAC sequencing datasets generated in this study have been deposited in the NCBI GEO database under accession code GSE221958. Code used to analyze datasets was generated from standard vignettes and is available from the corresponding author on reasonable request. Source data are provided with this paper.

## Source data

Source Data
