# Supplementary material for: Endothelial cell cycle state determines propensity for arterial-venous fate
Source: Nat Commun. 2022 Oct 6;13:5891. doi: 10.1038/s41467-022-33324-7 (PMC9537338; doi:10.1038/s41467-022-33324-7)
Supplement: Supplementary file 1 — Supplementary Information [file 41467_2022_33324_MOESM1_ESM.pdf]

Supplementary Figure 1

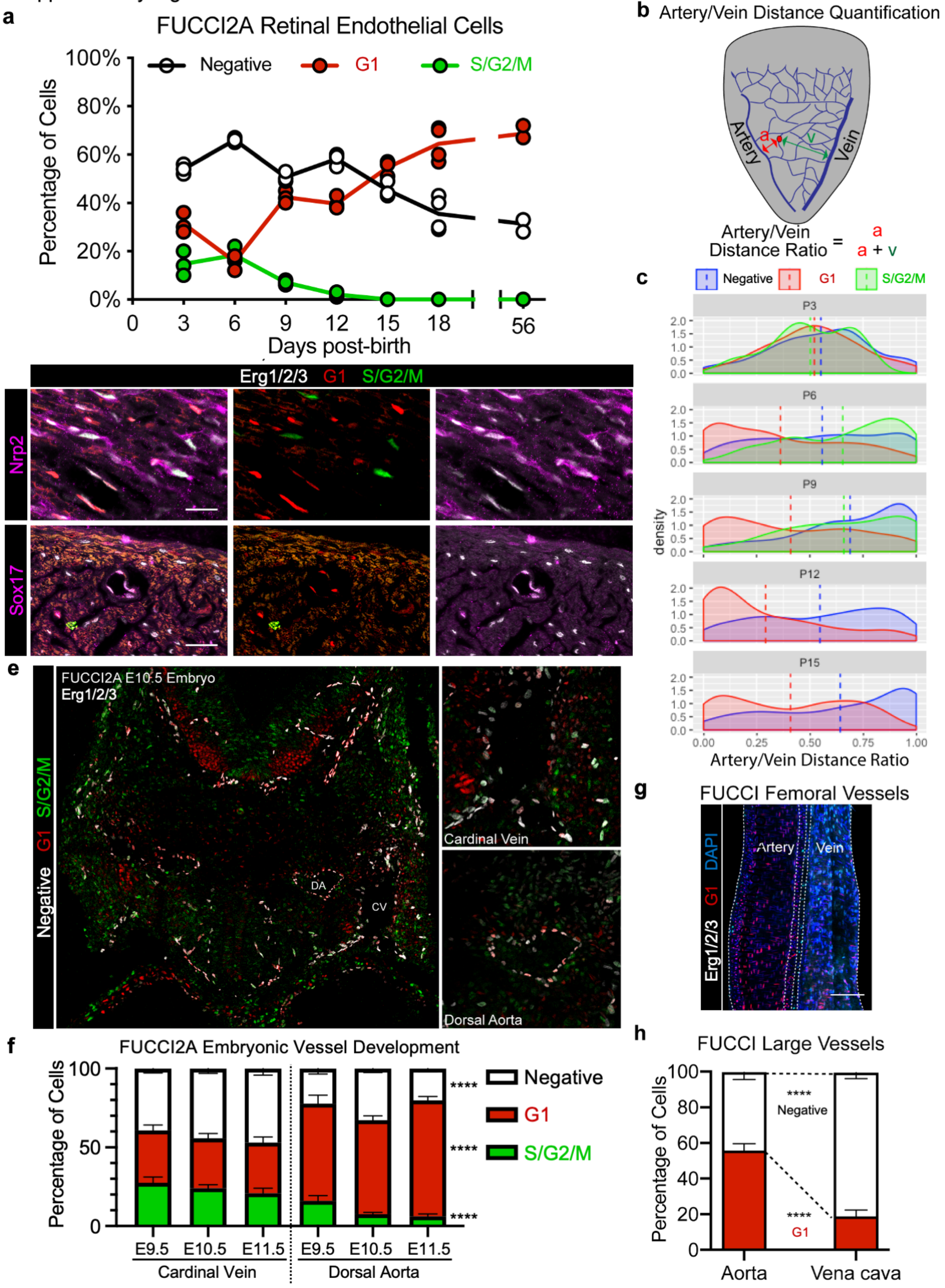

Supplementary Figure 1. Endothelial Cell Cycle State during Retinal Vascular Development. a, Cell cycle state of R26FUCCI2aR mouse retinal endothelial cells (experiment independently repeated three times). **b**, Overview of Artery/Vein Distance Ratio determination showing ratio calculated by shortest distance from cell to artery and divided by shortest distance to artery plus shortest distance to vein. **c**, Probability density of Artery/Vein Distance Ratio for FUCCI cell cycle states in retinal endothelial cells. **d**, Representative images of heart tissue sections from P6 Cdh5-Cre;R26FUCCI2aR mice immunostained with anti-Erg1/2/3 and (top panels) anti-Nrp2 or (bottom panels) anti-Sox17 (scale bars = 25 μm) **e**, Representative image of FUCCI2A E10.5 Embryo with magnified Cardinal Vein and Dorsal Aorta (experiment independently repeated four times using 3-4 embryos each experiment). **f**, Quantification of endothelial cells in FUCCI cell cycle states in the Cardinal Vein and Dorsal Aorta over E9.5, E10.5 and E11.5 (mean +/- SD, n = 12-15, p-values calculated by two-way ANOVA for Negative, G1 and S/G2/M). Endothelial cell cycle state in adult vessels shown by **g,** confocal z-stack imaged femoral vessels (experiment independently repeated three times), and **h,** quantified aorta and vena cava endothelial cells (mean +/- SD, n = 5, p-values calculated by two-way ANOVA post-hoc Tukey). Source data are provided as a Source Data file. Sample numbers derived from biological replicates of mice. Represented p-values as * < 0.05, ** < 0.01, *** < 0.001, **** < 0.0001.

Supplementary Figure 2

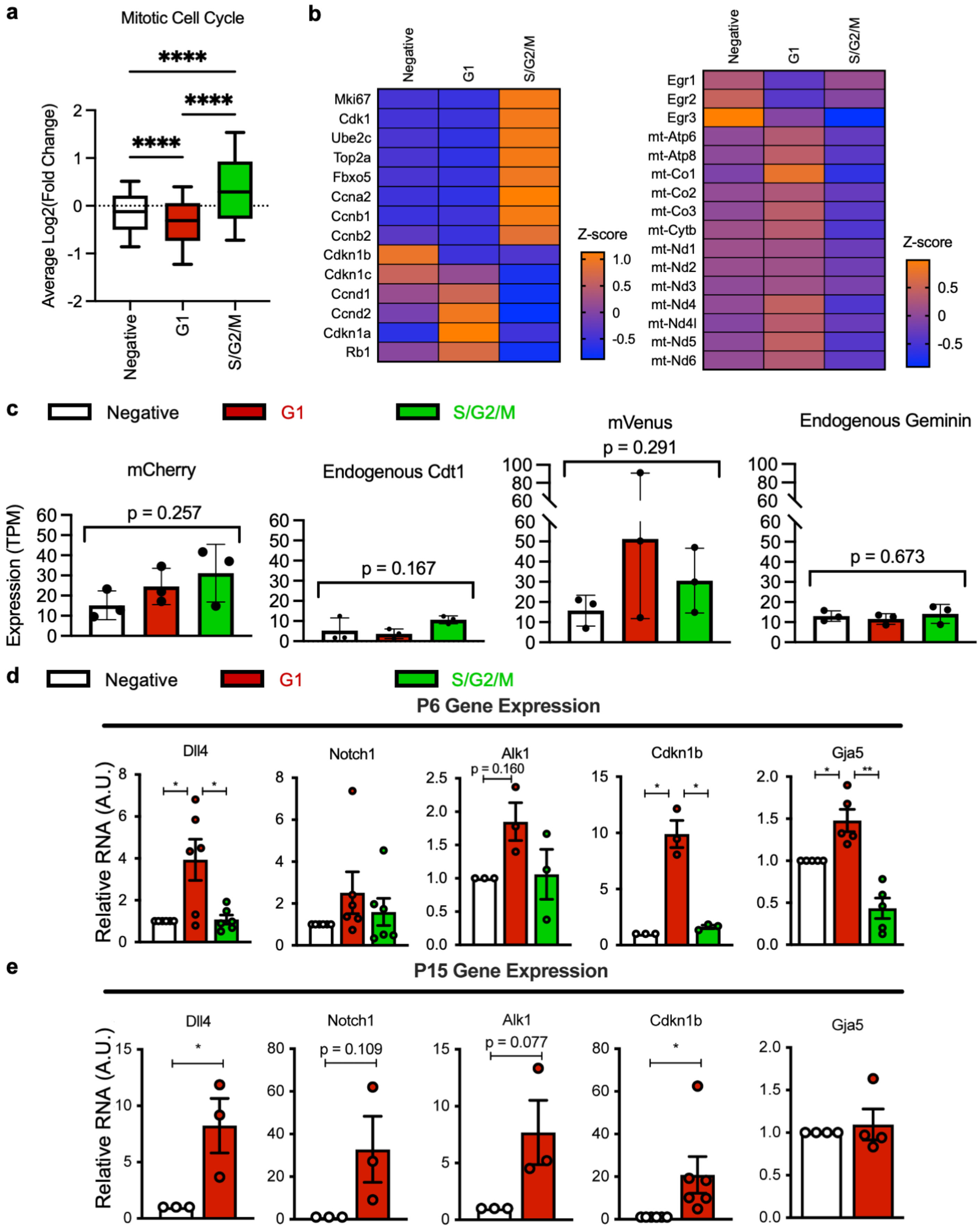

Supplementary Figure 2. Cell Cycle-Dependent Regulation of Gene Expression Analysis of Developing Retinal Endothelial Cells. **a**, Relative expression of genes associated with GO Term: Mitotic Cell Cycle in P6 retinal endothelial cell FUCCI population bulk RNA sequencing (n = 848 genes, box = 25th-75th percentile, center = median, whiskers = 10th-90th percentile). **b**, Relative expression of cell cycle-related, Egr1/2/3, and mitochondrial genes in P6 retinal endothelial cell FUCCI populations (average of n = 3, statistical test one-way ANOVA post-hoc Tukey). **c**, Expression in Transcripts per Million (TPM) within bulk RNA sequencing of FUCCI reporter fluorescent constructs and endogenous Cdt1 and Geminin (mean +/- SD, one-way ANOVA p-value reported). **d**,**e**, Gene expression by qRT-PCR of *Dll4*, *Notch1*, *Alk1*, *Cdkn1b*, and *Gja5* in retinal endothelial cells in cell cycle states at P6 and P15 (mean +/- SD, Statistical comparison of means by one-way ANOVA post-hoc Tukey for panel d or two-sided t-test for panel 3). Source data are provided as a Source Data file. Sample numbers derived from biological replicates of mice. Represented p-values as * < 0.05, ** < 0.01, *** < 0.001, **** < 0.0001.

Supplementary Figure 3

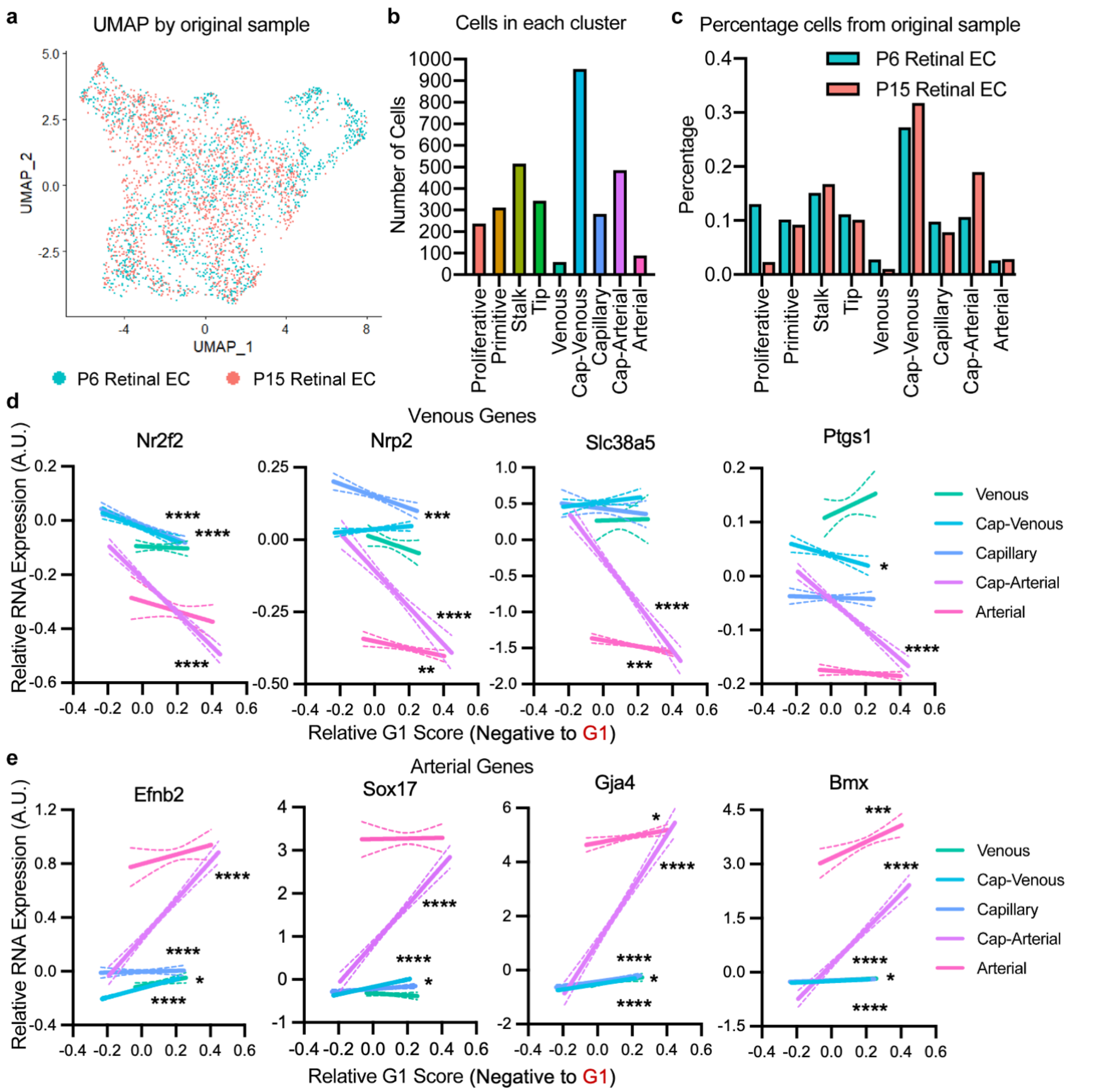

Supplementary Figure 3. Single Cell RNA Sequencing Analysis of Endothelial Cells during Retinal Vascular Development. a, UMAP dimensionality reduction plot of retinal endothelial cells from original P6 or P15 samples. **b**, Number of cells in each cluster. **c**, Percentage of cells from original P6 or P15 samples in each cluster. **d**,**e**, Linear regression of relative RNA expression compared to Negative score vs G1 Score in cells within venous, cap-venous, capillary, cap-arterial, and arterial for venous and arterial genes (statistical test of simple linear regression). Source data are provided as a Source Data file. Sample numbers derived from individual cells within the scRNAseq dataset. Represented p-values as * < 0.05, ** < 0.01, *** < 0.001, **** < 0.0001.

Supplementary Figure 4

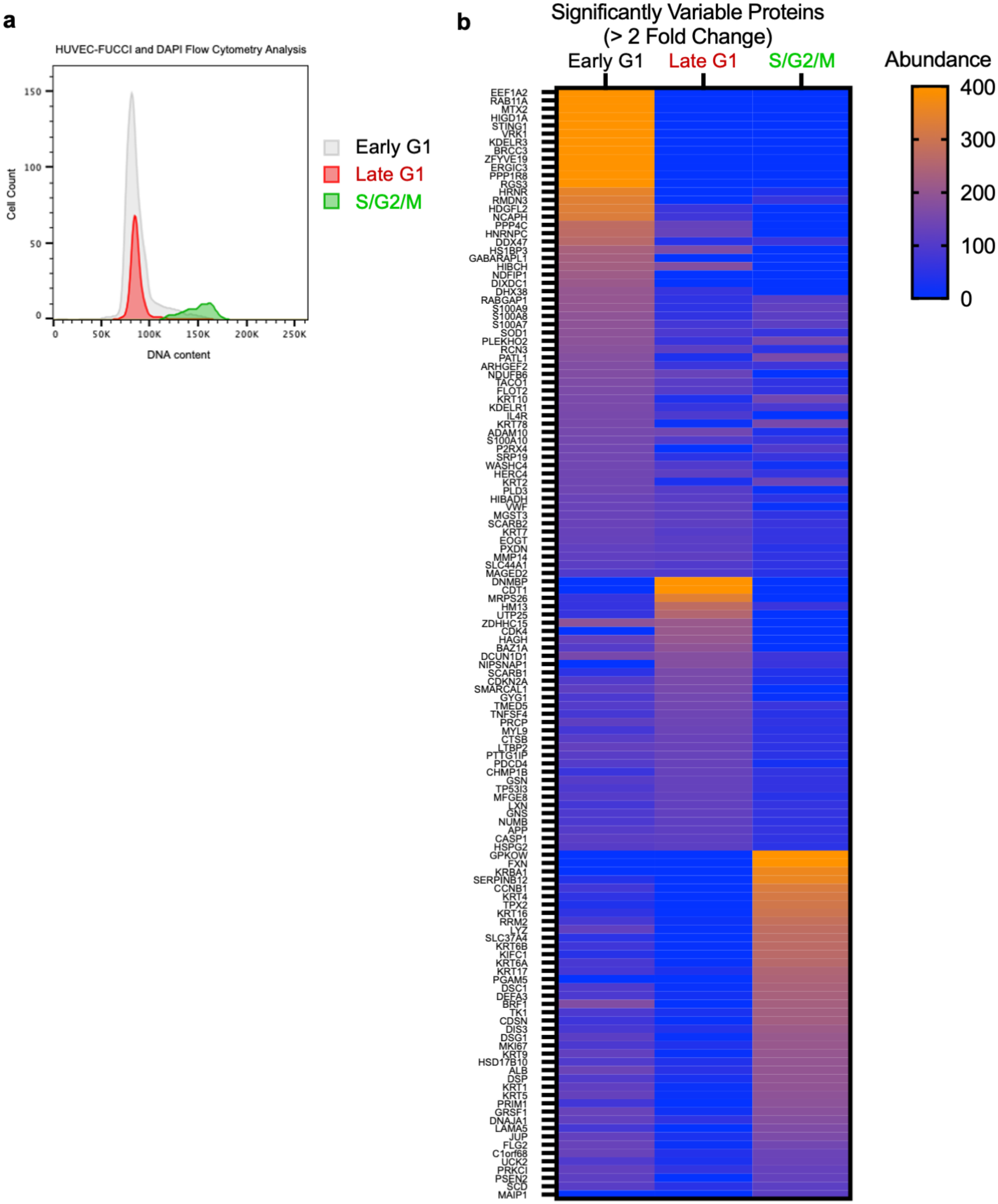

Supplementary Figure 4. Analysis of Cell Cycle States from HUVEC-FUCCI. a, Analysis of DNA content of HUVEC-FUCCI in Early G1, Late G1 and S/G2/M. **b**, All proteins identified by mass spectrometry of HUVEC-FUCCI cell cycle states with p-value < 0.05 and fold change > 2.0. Source data are provided as a Source Data file.

Supplementary Figure 5

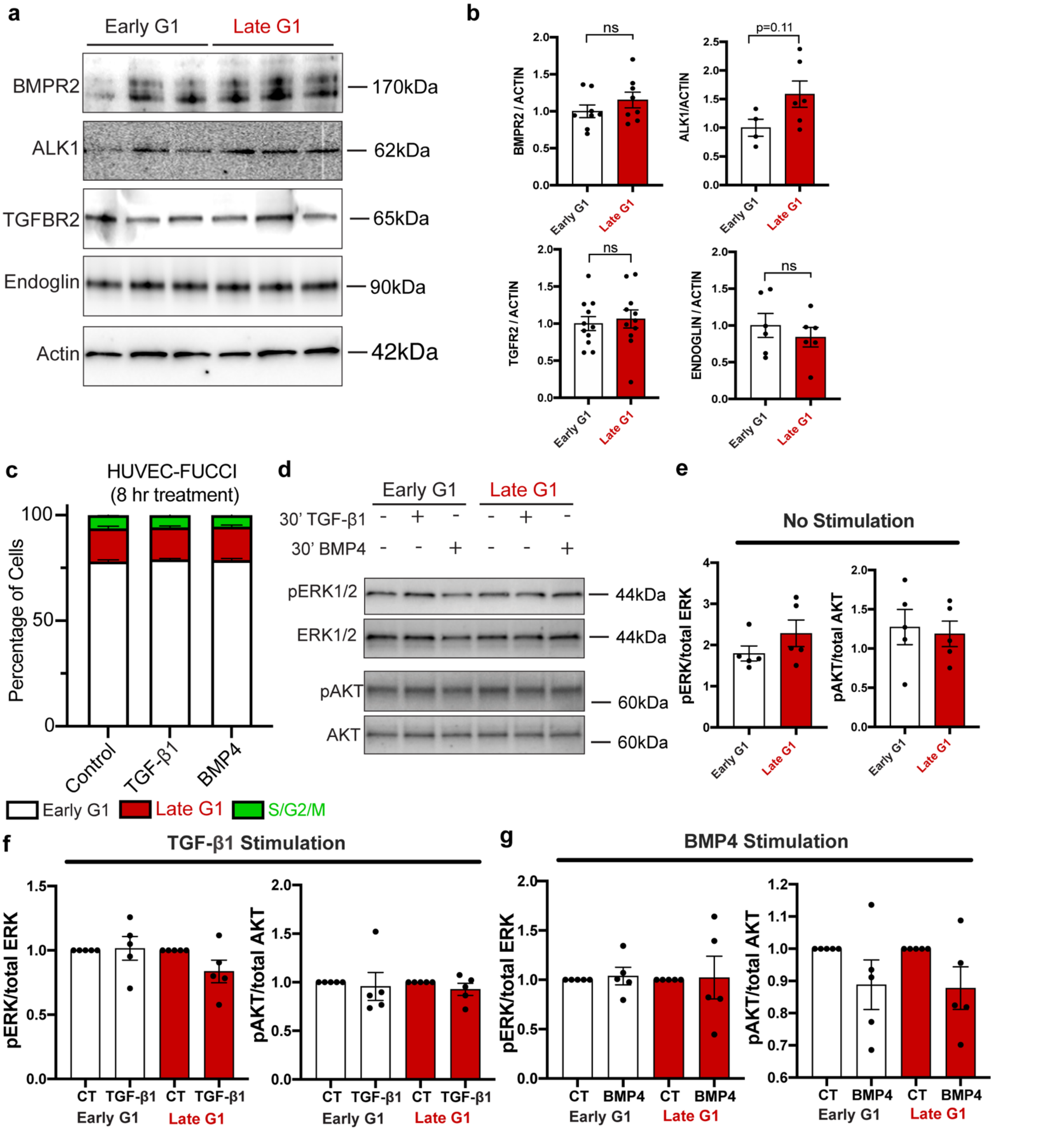

Supplementary Figure 5. Cell Cycle-dependent Expression and Induction of TGF-β /BMP Pathway in HUVEC-FUCCI. a,**b**, Representative western blot and quantification of TGF-β/BMP signaling proteins in HUVEC-FUCCI in early G1 and late G1 (mean +/- SEM, Statistical comparison of means by two-sided t-test). **c**,

HUVEC-FUCCI cell cycle states after TGF-β1 or BMP4 treatment (mean +/- SD, n = 3). **d-g**, Representative western blot and quantification of ERK1/2 and AKT phosphorylation in HUVEC-FUCCI in early G1 and late G1 after TGF-β1 or BMP4 treatment (mean +/- SEM, experiment independently repeated three times). Source data are provided as a Source Data file. Samples derived from biological replicates of HUVEC inductions. Represented p-values as * < 0.05, ** < 0.01, *** < 0.001, **** < 0.0001.

Supplementary Figure 6

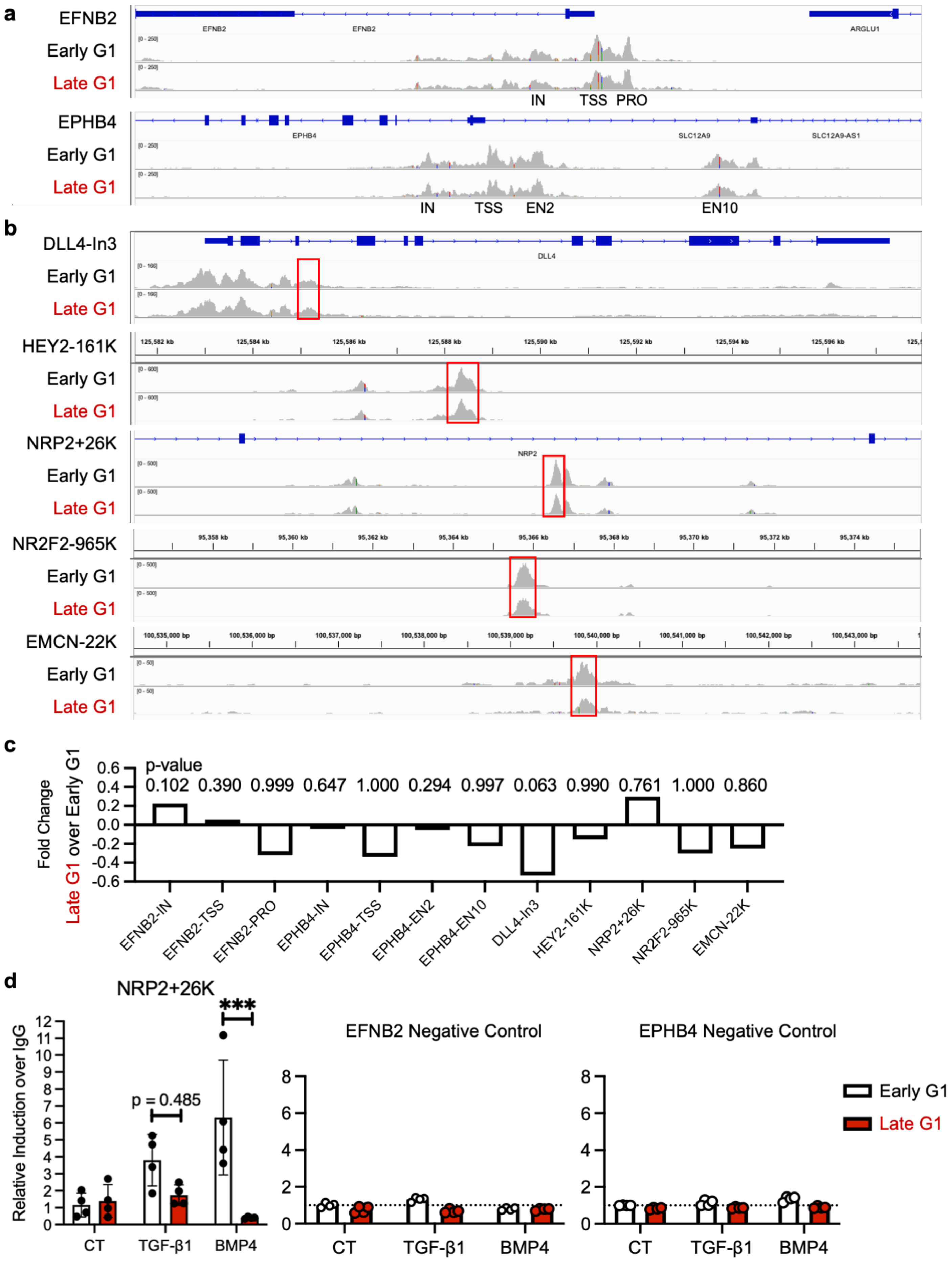

Supplementary Figure 6. ATAC Sequencing Analysis of Open Chromatin Peaks in HUVEC-FUCCI. a, Filtered ATAC sequencing reads near EFNB2 and EPHB4 locus with annotation. **b**, Filtered ATAC sequencing reads near known enhancer regions for arterial-venous gene expression: DLL4-In3, HEY2-161K, NRP2+26K, NR2F2-965K and EMCN-22K. **c**, Fold change (late G1 over early G1 for each peak with calculated p-value by computational analysis. **d**, ChIP-PCR of NRP2+26K and Negative control regions at 3000 bp upsteam of EFNB2-TSS (EFNB2 Negative Control) and 3000 bp upstream of EPHB4-EN2 (EPHB4 Negative Control), mean +/- SD, Statistical comparison of means by two-way ANOVA post-hoc Tukey. Sample numbers derived from biological replicates of HUVEC isolations. Source data are provided as a Source Data file. Represented p-values as * < 0.05, ** < 0.01, *** < 0.001, **** < 0.0001.

Supplementary Figure 7

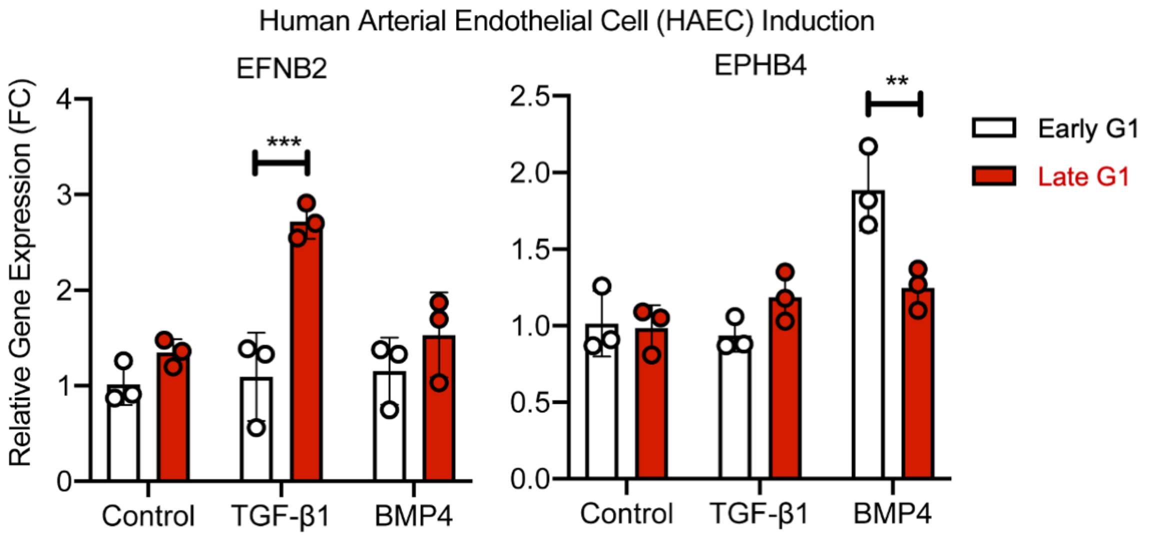

Supplementary Figure 7. TGF-β/BMP Induction of Arterial-venous Specification Genes in HAEC-FUCCI. Gene expression of EFNB2 and EPHB4 in HAEC-FUCCI after induction from TGF-β1 or BMP4 in early G1 or late G1 (mean +/- SD, n = 3). Source data are provided as a Source Data file. Sample numbers derived from biological replicates of HAEC inductions. Statistical comparison of means by two-way ANOVA post-hoc Tukey, represented p-values as * < 0.05, ** < 0.01, *** < 0.001, **** < 0.0001.

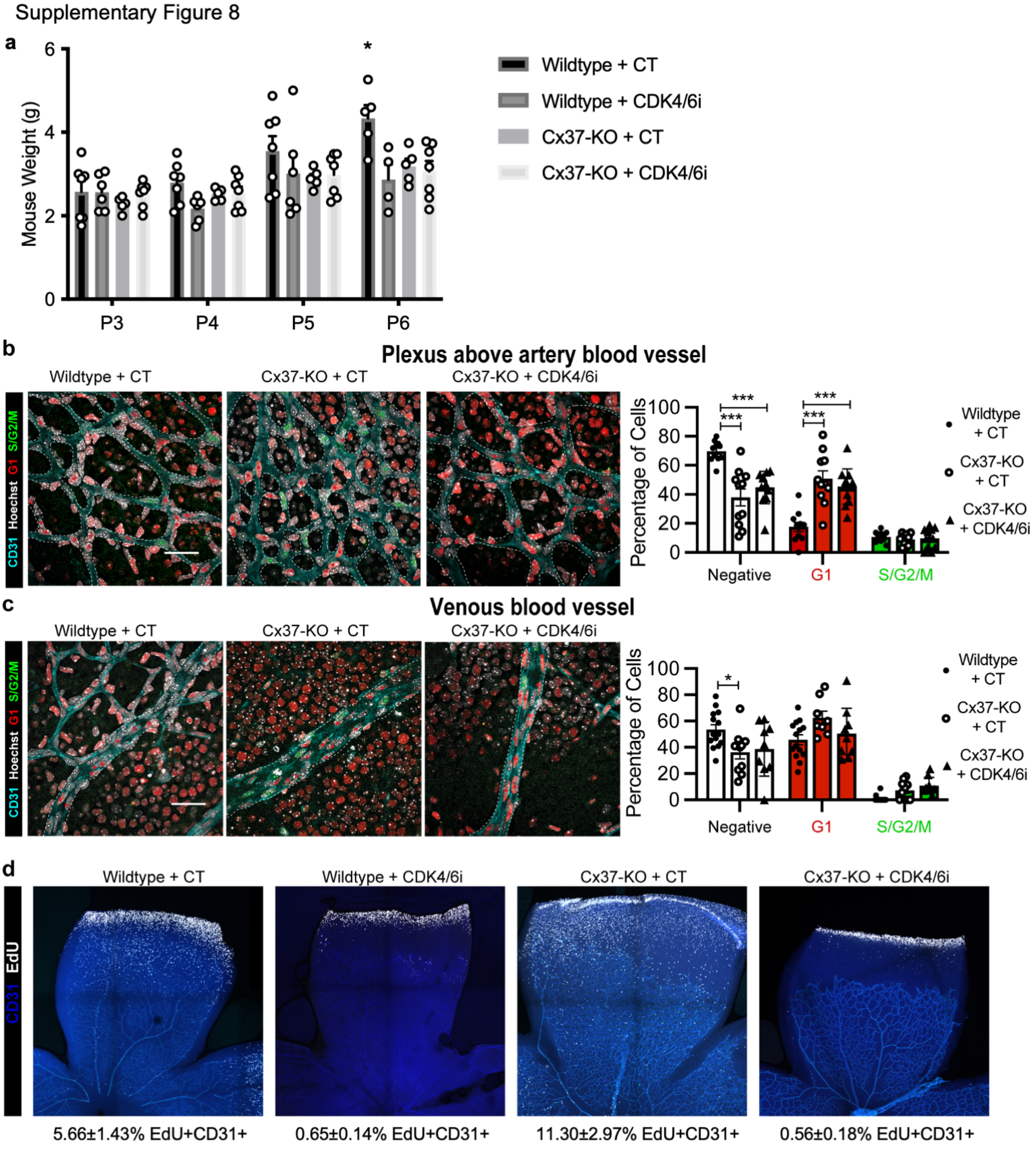

Supplementary Figure 8. Rescue of Arterial-venous Specification Defects with Pharmacological CDK4/5 Inhibition. a, WT, WT+CDK4/6i, Cx37-KO and Cx37-KO+CDK4/6i treated mice analyzed for weight over time (mean +/- SD, * indicates significant variability between other conditions at the same time point, Statistical comparison of means by two-way ANOVA post-hoc Tukey, p-value < 0.05). **b**,**c**, P6 retinal vasculature of R26p-FUCCI2, R26p-FUCCI2+Cx37-KO and R26p-FUCCI2+Cx37-KO+CDK4/6i treated mice imaged for CD31,

mVenus-hGem(1/110), mCherry-hCdt1(30/120) and Erg1/2/3 and quantified for cell cycle state in plexi above arterial blood vessels and venous blood vessels (mean +/- SD, scale bars = 50µm, vessels outlined in dotted white lines, cell cycle state highlighted with colored stars, experiment independently repeated three times, statistical test two-way ANOVA post-hoc Tukey). **d**, WT, WT+CDK4/6i, Cx37-KO and Cx37-KO+CDK4/6i treated mice analyzed for EdU incorporation and quantified for mean percentage with standard deviation of EdU+ cells of all CD31+ endothelial cells. Source data are provided as a Source Data file. Sample numbers derived from biological replicates of mice. Represented p-values as * < 0.05, ** < 0.01, *** < 0.001, **** < 0.0001.

Supplementary Table 1. Antibodies used in immunofluorescence, FACS, western blot and immunoprecipitation

Application	Antibody	Source	Dilution
Immunofluorescence	Goat anti-Mouse CD31	R&D Systems Cat# AF3628	1:100
	Rabbit anti-Mouse ERG1/2/3 (EPR3864)	AbCam Cat# ab92513	1:100
	Mouse anti-Mouse αSMA (1A4)	ThermoFisher Cat# 50-9760-82	1:100
	Goat anti-Mouse/Rat Neuropilin-2	R&D Systems Cat# AF567	1:100
	Goat anti-Human Sox17	R&D Systems Cat# AF1924	1:100
	Isolectin GS-IB4 Alexa Fluor 647	Invitrogen Cat# I32450	1:100
	SMAD2/3 (D7G7) Rabbit mAb	Cell Signaling Cat# 8685	1:100
	SMAD1 (D59D7) Rabbit mAb	Cell Signaling Cat# 6944	1:100
FACS	CD31-APC Rat anti-Mouse (MEC 13.3)	BD Biosciences Cat# 551262	1:100
	CD45-V450 Rag anti-Mouse (30-F11)	BD Biosciences Cat# 560501	1:100
Western Blot	Goat anti-TGFBR1	R&D Systems Cat# AF3025	1:1000
	Phospho-SMAD3 (C25A9) Rabbit mAb	Cell Signaling Cat# 9520	1:1000
	Phospho-SMAD1/5/9 (D5B10) Rabbit mAb	Cell Signaling Cat# 13820	1:1000
	B Actin (13E5) Rabbit mAb	Cell Signaling Cat# 4970	1:1000
	SMAD2/3 (D7G7) Rabbit mAb	Cell Signaling Cat# 8685	1:1000
	SMAD1 (D59D7) Rabbit mAb	Cell Signaling Cat# 6944	1:1000
	SMAD5 (D4G2) Rabbit mAb	Cell Signaling Cat# 12534	1:1000
	SMAD4 (D3R4N) Rabbit mAb	Cell Signaling Cat# 46535	1:1000
	Rabbit anti-BMPR2	AbCam Cat# ab96826	1:1000
	Goat anti-Human ALK1	R&D Systems Cat# AF370	1:1000
	Rabbit anti-TGF beta RII	AbCam Cat# ab186838	1:1000
	Goat anti-Human Endoglin	R&D Systems Cat# AF1097	1:1000
	Phospho p44/42 MAPK (Erk1/2) (E10)	Cell Signaling Cat# 9106	1:1000
	P44/42 MAPK (Erk1/2)	Cell Signaling Cat# 9102	1:1000
	Akt Rabbit Ab	Cell Signaling Cat# 9272	1:1000
	Phospho Akt (Ser473) (D9E) Rabbit mAb	Cell Signaling Cat# 4060	1:1000
	Horse anti-Goat IgG (H+L)	Vector Labs Cat# PI-9500	1:1000
	Goat anti-Rabbit IgG (H+L)	Vector Labs Cat# PI-1000	1:1000
Immunoprecipitation	SMAD4 (D3R4N) Rabbit mAb	Cell Signaling Cat# 46535	1:50

Supplementary Table 2. Genes enriched in arterial, capillary and venous endothelial cells.

Vascular Cell Identity	De la Paz & D'Amore, Cell Tiss Res 2009	Vanlandewijck et al., Nature 2018	Kalucka et al., Cell 2020	Crist et al., Gene Expr Patterns 2017
Venous	Ephb4	Slc38a5	Apoe	Apj
	Nr2f2		Bgn	Flt4
	Nrp2		Ctla2a	
	Emcn		Il6st	
			Ptgs1	
			Tmsb10	
Capillary	Kdr	Mfsd2a	Rgcc	
		Ca1	Sgk1	
			Sparc	
Arterial	Efrnb2	Bmx	Clu	Jag1
	Gja5	Gkn3	Crip1	Dll4
	Hey1		Fbln1	
	Hey2		Mecom	
	Nrp1		Sat1	
	Sox17		Sema3g	
	Alk1			

Supplementary Table 3. Gene-specific primers for qRT-PCR

Gene	Forward Primer (5'-3')	Reverse Primer (5'-3')
Mouse Efnb2	GTGCCAGACAAGAGCCATGAA	GGTGCTAGAACCTGGATTTGG
Human EFNB2	TATGCAGAACTGCGATTTCCAA	TGGGTATAGTACCAGTCCTTGTC
Mouse Gja4	CCCACATCCGATACTGGGTG	CGAAGACGACCGTCCTCTG
Human GJA4	ACACCCACCCTGGTCTACC	CACTGGCGACATAGGTGCC
Mouse Gja5	CCACAGTCATCGGCAAGGTC	CTGAATGGTATCGCACCGGAA
Human GJA5	CCGTGGTAGGCAAGGTCTG	ATCACACCGGAAATCAGCCTG
Mouse Hey2	AAGCGCCCTTGTGAGGAAAC	GGTAGTTGTCGGTGAATTGGAC
Mouse Sox17	CTTTATGGTGTGGGCCAAAGA	CTTCCAAGACTTGCCTAGCATC
Human SOX17	AGCGCCCTTCACGTGTACTA	CTTGCACACGAAGTGCAGAT
Mouse Ephb4	CACCCAGCAGCTTGATCCTG	ACCAGGACCACACCCACAAC
Human EPHB4	CGCACCTACGAAGTGTGTGA	GTCCGCATCGCTCTCATAGTA
Mouse Nr2f2	ATGTAGCCCATGTGGAAAGC	CCTACCAAACGGACGAAAAA
Human NR2F2	GGACCACATACGGATCTTCCAA	ACATCAGACAGACCACAGGCAT
Mouse Nrp2	CGGCTCTGACATCCACATAG	CTCGCTCGCGATCTCTG
Human NRP2	GTGGTTCATCTTGACCTTGT	ATTCTTCTTCTGCAACCTCA
Mouse Ptgs1	CAGTGCGGTCCAACCTTAT	GAGGGCAGAATGCGAGTATAG
Mouse Dll4	GGAACCTTCTCACTCAACATCC	CTCGTCTGTTCGCCAAATCT
Mouse Notch1	TATGGCCACGAGGAAGAGCT	TAGACAATGGAGCCACGGATG
Mouse Alk1	TGACCTCAAGAGTCGCAATG	CTCGGGTGCCATGTATCTTT
Mouse Cdkn1b	TCAAACGTGAGAGTGTCTAACG	CCGGGCCGAAGAGATTTCTG
Human SMAD1	ACCTGCTTACCTGCCTCCTG	CATAAGCAACCGCCTGAACA
Human SMAD2	ACCGAAATGCCACGGTAGAA	TGGGGCTCTGCACAAAGAT
Human SMAD3	CCTGAGTGAAGATGGAGAAACC	GGCTGCAGGTCCAAGTTATTA
Human SMAD5	AGCCTTCTGGTTCAGTTTAGG	AAGGGCTGTTTGGAGATAAGG
Mouse Actb	AGAGGGAAATCGTGCGTGAC	CAATAGTGATGACCTGGCCGT
Human ACTB	TCACCCACACTGTGCCCATCTACGA	CAGCGGAACCGCTCATTGCCAATGG
ATAC-Seq Peaks		
EFNB2_PRO	GTCGAGCGCCGGTGCTGAC	CGCAGAGCGCTCGAGATGCG
EFNB2_TSS	CAG AGC GCT ATA AAC GCG GGG	GACGGACCGGCCGTCCAAC
EFNB2_INT	GTTACTCTC CACAAGGCCTGGCTC	CTTCTCCCTCGGGACCCTGGTAG
EPHB4_EN10	GCTGGCTCTAGGAGGGAAAGTAAGAAG	GCTTCCCTAACCAGCCCAAGAGCTTC
EPHB4_EN2	CTCCTCCCTCCCCGATGCAG	CAAAAGGCAGGGCAGGGGTTTGG
EPHB4_TSS	CATGAGGGAAGCGGAGAGGGGC	GTTCCTCACCCGCCGCCC
EPHB4_INT	CTCCATTGTTCCAGCCGGGATAGTG	GAAAGGGAGCCTGCGCTTGC
NRP2_26K	CTCCACTGGTCGCTACAATA	CTGTGTGAGTCAGACCTTGA
EFNB2_CD	CAGACTGATTTGATACAGACATACA	AGAGGAGATCCGTTACGATTG
EPHB4_CD	ATCGAGACCATCCTGGCTAA	ACTACAGGCACCCACCA

Supplementary Table 4. Gene Ontology analysis of variable peaks within ATAC sequencing datasets of HUVEC-FUCCI in early G1 and late G1 cell cycle states

GO Term	p-value	Fold Enrichment
Early G1		
regulation of epithelial cell migration	4.56E-04	1.42
cell cycle phase transition	1.02E-04	1.41
mitotic cell cycle phase transition	1.44E-04	1.4
actin cytoskeleton organization	3.46E-06	1.36
angiogenesis	4.05E-04	1.35
mitotic cell cycle process	3.92E-07	1.34
blood vessel morphogenesis	8.96E-05	1.34
positive regulation of cell cycle process	1.03E-03	1.34
negative regulation of organelle organization	5.72E-04	1.34
regulation of supramolecular fiber organization	2.55E-04	1.34
regulation of mitotic cell cycle phase transition	1.12E-04	1.33
blood vessel development	2.12E-05	1.33
vasculature development	2.63E-05	1.32
regulation of small GTPase mediated signal transduction	9.50E-04	1.32
organelle localization	6.03E-06	1.32
positive regulation of cell cycle	3.39E-04	1.32
Late G1		
proteoglycan metabolic process	6.30E-04	1.77
cardiac chamber morphogenesis	1.33E-04	1.71
neuron projection guidance	9.36E-08	1.66
axon guidance	1.16E-07	1.66
regulation of BMP signaling pathway	1.21E-03	1.65
negative regulation of cellular response to growth factor stimulus	1.25E-03	1.63
regulation of cell-matrix adhesion	9.08E-04	1.62
cardiac chamber development	8.46E-05	1.62
neuron migration	1.02E-03	1.6
limb morphogenesis	3.68E-04	1.59
appendage morphogenesis	3.68E-04	1.59
cardiac ventricle development	1.08E-03	1.59
morphogenesis of a branching structure	2.03E-04	1.59
morphogenesis of a branching epithelium	3.03E-04	1.59
regulation of tube size	5.90E-04	1.59
branching morphogenesis of an epithelial tube	1.01E-03	1.59
