# Supplementary material for: Endothelial cell cycle state determines propensity for arterial-venous fate
Source: Nat Commun. 2022 Oct 6;13:5891. doi: 10.1038/s41467-022-33324-7 (PMC9537338; doi:10.1038/s41467-022-33324-7)
Supplement: Supplementary file 2 — Description of Additional Supplementary Files [file 41467_2022_33324_MOESM2_ESM.pdf]

Description of Additional Supplementary Files

Supplementary Movie 1. PHATE plot 3D with overlaid clusters and cell cycle scores

Supplementary Movie 2. Live-cell imaging of proliferating HUVEC-FUCCI over 48 hours

Supplementary Data 1. Gene expression results from bulk RNA sequencing of P6 R26FUCCI2aR retinal endothelial cells in FUCCI-Green S/G2/M, FUCCI-Negative and FUCCI-Red G1 cell cycle states

Supplementary Data 2. Gene expression results from single cell RNA sequencing of P6 and P15 retinal endothelial cells.

Supplementary Data 3. Individual cell scoring from single cell RNA sequencing dataset.

Supplementary Data 4. Protein expression results from mass spectrometry analysis of HUVEC-FUCCI in S/G2/M, early G1, and late G1 cell cycle states

Supplementary Data 5. Gene expression results from bulk RNA sequencing of HUVEC-FUCCI in S/G2/M, early G1, late G1, and G1/S cell cycle states

Supplementary Data 6. Peak quantification results from ATAC sequencing of early G1 and late G1 HUVEC-FUCCI
