# Supplementary material for: Endothelial cell cycle state determines propensity for arterial-venous fate
Source: Nat Commun. 2022 Oct 6;13:5891. doi: 10.1038/s41467-022-33324-7 (PMC9537338; doi:10.1038/s41467-022-33324-7)
Supplement: Supplementary file 11 — Reporting Summary [file 41467_2022_33324_MOESM11_ESM.pdf]

nature portfolio

Corresponding author(s): Hirschi, Karen

Last updated by author(s): 8/22/2022

Reporting Summary

Nature Portfolio wishes to improve the reproducibility of the work that we publish. This form provides structure for consistency and transparency in reporting. For further information on Nature Portfolio policies, see our Editorial Policies and the Editorial Policy Checklist.

Statistics

For all statistical analyses, confirm that the following items are present in the figure legend, table legend, main text, or Methods section.

n/a	Confirmed	
☐	☒	The exact sample size (n) for each experimental group/condition, given as a discrete number and unit of measurement
☐	☒	A statement on whether measurements were taken from distinct samples or whether the same sample was measured repeatedly
☐	☒	The statistical test(s) used AND whether they are one- or two-sided<br>*Only common tests should be described solely by name; describe more complex techniques in the Methods section.*
☐	☒	A description of all covariates tested
☐	☒	A description of any assumptions or corrections, such as tests of normality and adjustment for multiple comparisons
☐	☒	A full description of the statistical parameters including central tendency (e.g. means) or other basic estimates (e.g. regression coefficient) AND variation (e.g. standard deviation) or associated estimates of uncertainty (e.g. confidence intervals)
☐	☒	For null hypothesis testing, the test statistic (e.g. F, t, r) with confidence intervals, effect sizes, degrees of freedom and P value noted<br>*Give P values as exact values whenever suitable.*
☐	☒	For Bayesian analysis, information on the choice of priors and Markov chain Monte Carlo settings
☐	☒	For hierarchical and complex designs, identification of the appropriate level for tests and full reporting of outcomes
☒	☐	Estimates of effect sizes (e.g. Cohen's d, Pearson's r), indicating how they were calculated

Our web collection on statistics for biologists contains articles on many of the points above.

Software and code

Policy information about availability of computer code

Data collection	All software used for data collection is described within the methods regarding the sample collection. No novel software was generated for these studies. RNA sequencing data was collected using Illumina software.
Data analysis	All software used for data analysis is described within the methods regarding the sample collection. No novel software was generated for these studies. RNA sequencing data was analyzed using FastQC, v0.11.9, Kallisto v0.46.2, GAGE v2.40.2, Seurat v3.0, PHATE v0.3.0, and MAGIC v3.0.0. Mass Spectrometry data was analyze using Proteome Discoverer (PD) software version 2.4.0.305. ATAC sequencing data was analyzed using FastQC v0.11.9, Trimmomatic v0.33, MACS2 v2.2.7.1, and HOMER v4.11.

For manuscripts utilizing custom algorithms or software that are central to the research but not yet described in published literature, software must be made available to editors and reviewers. We strongly encourage code deposition in a community repository (e.g. GitHub). See the Nature Portfolio guidelines for submitting code & software for further information.

Data

Policy information about availability of data

All manuscripts must include a data availability statement. This statement should provide the following information, where applicable:

- Accession codes, unique identifiers, or web links for publicly available datasets
- A description of any restrictions on data availability
- For clinical datasets or third party data, please ensure that the statement adheres to our policy

Source data are provided as a Source Data file. The bulk RNA sequencing datasets generated in this study have been deposited in the NCBI GEO database under accession code GSE211658 [https://www.ncbi.nlm.nih.gov/geo/query/acc.cgi?acc=GSE211658]. The single cell RNA sequencing dataset of P6 and P15 retinal endothelial cells generated in this study has been deposited in the NCBI GEO database under accession code GSE169039 [https://www.ncbi.nlm.nih.gov/geo/query/

acc.cgi?acc=GSE169039]. Proteomics datasets generated in this study have been deposited in the PRIDE database under reference code 1-20220824-53717. The bulk ATAC sequencing datasets generated in this study have been deposited in the NCBI GEO database under accession code GSE221958 [https://www.ncbi.nlm.nih.gov/geo/query/acc.cgi?acc=GSE211958].

Field-specific reporting

Please select the one below that is the best fit for your research. If you are not sure, read the appropriate sections before making your selection.

- [x] Life sciences
- [ ] Behavioural & social sciences
- [ ] Ecological, evolutionary & environmental sciences

For a reference copy of the document with all sections, see nature.com/documents/nr-reporting-summary-flat.pdf

Life sciences study design

All studies must disclose on these points even when the disclosure is negative.

Sample size	Sample sizes of experiments were determined based on sample sizes from previous similar experiments (Fang et al., Nature Communications 2017) and sample availability.
Data exclusions	Sequencing data were excluded as inaccurately sequenced cells or contaminating cells. These exclusion criteria were: RNA counts < 200, RNA counts > 3500, mitochondrial percentage < 5%, expression of Hbb.bt (Blood cells), Neurod1 (Neural cells), or Cd63 (Pericytes). No data from molecular experiments were excluded except for human error.
Replication	All experiments were replicated by multiple biological replicates, and several mouse FUCCI experiments were replicated in multiple mouse lines. All replication experiments successfully supported the results in the manuscript.
Randomization	Randomization is not applicable to our studies because treatment groups used entire mouse litters for analysis.
Blinding	Objective analysis and quantification was obtained or confirmed by researchers blinded to the sample groups.

Reporting for specific materials, systems and methods

We require information from authors about some types of materials, experimental systems and methods used in many studies. Here, indicate whether each material, system or method listed is relevant to your study. If you are not sure if a list item applies to your research, read the appropriate section before selecting a response.

Materials & experimental systems

n/a	Involved in the study	
[ ]	[x]	Antibodies
[ ]	[x]	Eukaryotic cell lines
[x]	[ ]	Palaeontology and archaeology
[ ]	[x]	Animals and other organisms
[x]	[ ]	Human research participants
[x]	[ ]	Clinical data
[x]	[ ]	Dual use research of concern

Methods

n/a	Involved in the study	
[x]	[ ]	ChIP-seq
[ ]	[x]	Flow cytometry
[x]	[ ]	MRI-based neuroimaging

Antibodies

Antibodies used

Immunofluorescence

Goat anti-Mouse CD31 R&D Systems Cat# AF3628
Rabbit anti-Mouse ERG1/2/3 (EPR3864) AbCam Cat# ab92513
Mouse anti-MouseSMA (1A4) ThermoFisher Cat# 50-9760-82
Goat anti-Mouse/Rat Neuropilin-2 R&D Systems Cat# AF567
Goat anti-Human Sox17 R&D Systems Cat# AF1924
Isolectin GS-IB4 Alexa Fluor 647 Invitrogen Cat# I32450
SMAD2/3 (D7G7) Rabbit mAb Cell Signaling Cat# 8685
SMAD1 (D59D7) Rabbit mAb Cell Signaling Cat# 6944

FACS

CD31-APC Rat anti-Mouse (MEC 13.3) BD Biosciences Cat# 551262
CD45-V450 Rag anti-Mouse (30-F11) BD Biosciences Cat# 560501

	Western Blot<br><br>Goat anti-TGFBR1 R&D Systems Cat# AF3025<br>Phospho-SMAD3 (C25A9) Rabbit mAb Cell Signaling Cat# 9520<br>Phospho-SMAD1/5/9 (D5B10) Rabbit mAb Cell Signaling Cat# 13820<br>B Actin (13E5) Rabbit mAb Cell Signaling Cat# 4970<br>SMAD2/3 (D7G7) Rabbit mAb Cell Signaling Cat# 8685<br>SMAD1 (D59D7) Rabbit mAb Cell Signaling Cat# 6944<br>SMAD5 (D4G2) Rabbit mAb Cell Signaling Cat# 12534<br>SMAD4 (D3R4N) Rabbit mAb Cell Signaling Cat# 46535<br>Rabbit anti-BMPR2 AbCam Cat# ab96826<br>Goat anti-Human ALK1 R&D Systems Cat# AF370<br>Rabbit anti-TGF beta RII AbCam Cat# ab186838<br>Goat anti-Human Endoglin R&D Systems Cat# AF1097<br>Phospho p44/42 MAPK (Erk1/2) (E10) Cell Signaling Cat# 9106<br>P44/42 MAPK (Erk1/2) Cell Signaling Cat# 9102<br>Akt Rabbit Ab Cell Signaling Cat# 9272<br>Phospho Akt (Ser473) (D9E) Rabbit mAb Cell Signaling Cat# 4060<br>Horse anti-Goat IgG (H+L) Vector Labs Cat# PI-9500<br>Goat anti-Rabbit IgG (H+L) Vector Labs Cat# PI-1000<br><br>Immunoprecipitation<br><br>SMAD4 (D3R4N) Rabbit mAb Cell Signaling Cat# 46535
Validation	All antibodies have been validated by companies generating the antibodies. Experimental validations were performed through IgG controls or internal experimental controls.<br>Validation Statements from Companies:<br>Cell Signaling Technologies: To ensure our antibodies will work in your experiment, we adhere to the Hallmarks of Antibody Validation™, six complementary strategies that can be used to determine the functionality, specificity, and sensitivity of an antibody in any given assay. CST adapted the work by Uhlen, et. al., ("A Proposal for Validation of Antibodies." Nature Methods (2016)) to build the Hallmarks of Antibody Validation, based on our decades of experience as an antibody manufacturer and our dedication to reproducible science.<br>AbCam: To achieve accurate and precise results, you need antibodies that consistently bind specifically and selectively to the intended target. Antibody validation must be application-specific to be effective and information on which applications an antibody has been validated in can be found in the Tested Applications section on any antibody datasheet.<br>BD Biosciences: The specificity is confirmed using multiple methodologies that may include a combination of flow cytometry, immunofluorescence, immunohistochemistry or wester blot to test staining on a combination of primary cells, cell lines or transfectant models. All flow cytometry reagents are titrated on the relevant positive or negative cells.<br>ThermoFisher Invitrogen: Invitrogen antibodies are currently undergoing a rigorous two-part testing approach. Part 1—Target specificity verification. This helps ensure the antibody will bind to the correct target. Part 2—Functional application validation<br>These tests help ensure the antibody works in a particular application(s) of interest.<br>R&D Systems: With the recent emergence of antibody reproducibility being questioned in many headlines, it is comforting to know that R&D Systems has taken antibody validation and reproducibility seriously for over 30 years. Our highly selective development and validation criteria ensure only the best antibodies end up in your hands. Antibody validation process: Antigen Design, Feasibility Testing, Specificity and Sensitivity Testing, Release Decision and Quality Control.

Eukaryotic cell lines

Policy information about cell lines

Cell line source(s)	HUVEC: Human Umbilical Vein Endothelial Cells - Yale Vascular Biology and Therapeutics Core<br>HAEC: Human Aortic Endothelial Cells - Lonza, Cat# CC-2535
Authentication	Cell sources are both primary cell lines authenticated through the organization from which they were purcased.
Mycoplasma contamination	All cell lines tested negative for Mycoplasma.
Commonly misidentified lines (See ICLAC register)	No commonly misidentified lines were used in this study.

Animals and other organisms

Policy information about studies involving animals; ARRIVE guidelines recommended for reporting animal research

Laboratory animals	Mouse - Cdh5-CreERT2 x R26FUCCI2aR - M/F - P3 to 8 weeks<br>Mouse - Gja4-/- x R26p-FUCCI2 - M/F - P6
Wild animals	No wild animals were used in this study.

Field-collected samples: No field animals were used in this study.

Ethics oversight: All animal procedures were approved by the Institute for Animal Care and Use Committees at Yale University and the University of Virginia.

Note that full information on the approval of the study protocol must also be provided in the manuscript.

Flow Cytometry

Plots

Confirm that:

- [x] The axis labels state the marker and fluorochrome used (e.g. CD4-FITC).
- [x] The axis scales are clearly visible. Include numbers along axes only for bottom left plot of group (a 'group' is an analysis of identical markers).
- [x] All plots are contour plots with outliers or pseudocolor plots.
- [x] A numerical value for number of cells or percentage (with statistics) is provided.

Methodology

Sample preparation: Primary murine retinal endothelial cells were digested and isolated as previously described (Chavkin et al, J Vasc Res 2021). HUVEC-FUCCI were grown in cell culture and isolated through trypsin digestion before sorting.

Instrument: BD FACSAria
BD FACSMelody

Software: FlowJo, Version 10.6.1.

Cell population abundance: Lowest cell populations were 0.5% of main population, but most populations were >10% of total population. For 0.5% population: endothelial cells isolated from murine retinal tissue yields low abundance, but we performed optimization and confirmation of this population to confirm accuracy (Chavkin et al, J Vasc Res 2021).

Gating strategy: Primary murine retinal endothelial cells: FSC-A/SSC-A -> FSC-H/FSC-A -> PI+ -> CD31+/CD45- -> Sort on GFP/RFP
(Gating strategy and controls are published in Chavkin et al, J Vasc Res 2021)
HUVEC-FUCCI: FSC-A/SSC-A -> FSC-H/FSC-A -> Sort on GFP/RFP
(Gating strategy for FUCCI reporter is presented in Figure 5B)

- [x] Tick this box to confirm that a figure exemplifying the gating strategy is provided in the Supplementary Information.
